# Computational Multieffect Analysis of Blood‐Based Trihybrid Nanofluid Flow With Nanoparticles Through a Stenotic Artery

**DOI:** 10.1155/bmri/6374799

**Published:** 2026-08-14

**Authors:** Muhammad Sajjad Hossain, Mashrur Mahfuz, Mahtab Uddin, Ashek Ahmed, Kazi Ekramul Hoque, Golam Mostafa

**Affiliations:** ^1^ Department of Arts and Sciences, Ahsanullah University of Science and Technology, Dhaka, Bangladesh, aust.edu; ^2^ Department of Mechanical and Production Engineering, Ahsanullah University of Science and Technology, Dhaka, Bangladesh, aust.edu; ^3^ Institute of Natural Sciences, United International University, Dhaka, Bangladesh, uiu.ac.bd; ^4^ Department of Computer Science and Engineering, Southeast University, Dhaka, Bangladesh, seu.edu.bd

**Keywords:** cardiovascular disease modeling, finite element method, therapeutic flow management, thrombosis risk prediction, trihybrid nanoparticle

## Abstract

This study is aimed at examining the computational modeling of the blood flow of a trihybrid nanofluid with nanoparticles such as Ag, Au, and Cu to simulate the hemodynamics parameters and heat transfer within a constricted channel geometry that directly parallels the flow conditions in a stenosed artery. The flow is defined as an unsteady, incompressible, laminar Newtonian fluid, with blood exhibiting Newtonian properties at elevated shear rates. An algorithm for mass, velocity, and energy, as well as a fine element‐sized mesh, was developed using the computational fluid dynamics (CFD) technique based on the finite element method. Here, systematic observation of the effect of varying nanoparticle volume fractions (*ϕ* = 3*%*, 9*%*, 15*%*) and Reynolds numbers (Re) on the resulting flow velocity profiles. Although maximal velocity is geometrically concentrated at the stenosis throat, the critical determining factor of patient danger is the concentration‐dependent change in fluid viscosity. Increasing *ϕ* raises effective viscosity (Brinkman model), progressively flattening the velocity profile into a blunt, plug‐like regime; paradoxically, this near‐uniform plug, lacking the gradual wall‐to‐core velocity gradient of a parabolic profile, is unable to resist the adverse pressure gradient at the abrupt poststenotic expansion, causing simultaneous cross‐sectional deceleration and abrupt flow separation that sustains larger, more energetic recirculation zones than those observed at lower *ϕ*. This disturbed distal flow that generates large and persistent recirculation zones downstream of the stenosis, which are associated with increased thrombotic risk due to disturbed flow patterns. Conversely, the increase in *ϕ* drastically increases thermal conductivity, making the heat transfer process nearly independent of flow speed, that a key insight for forthcoming contained thermal therapies. Eventually, this research delivers a mechanistic link between fluid rheology, flow instability, and thrombotic risk, signifying that therapeutic strategies need to prioritize the management of fluid viscosity to stabilize poststenotic flow fields and mitigate associated cardiovascular complications. This work is the first effort to evaluate the impact of trihybrid nanofluid rheology on the exacerbation of poststenotic flow instability at clinically relevant Re. This reveals that a critical higher nanoparticle loading (*ϕ*) improves thermal conductivity. The present findings have direct significance for biomedical applications like targeted photothermal therapy (PTT), antimicrobial vascular intervention, and nanoparticle‐mediated drug delivery in cardiovascular disease management. The computed 18% enhancement in conductive heat flux at *ϕ*_*T*_ = 15*%* further establishes a quantitative performance baseline for the design of nanofluid‐based thermal management systems in biomedical microdevices.

## 1. Introduction

The circulatory system is primarily responsible for removing waste materials from the body and delivering essential nutrients and oxygen to the active tissues through blood. It is a puzzling network of blood vessels. Several studies have been directed to further clarify the phenomena of blood flow in arteries. According to Chandran et al. [1], the fundamental parameters and phenomena that fully characterize the blood flow phenomenon are velocity, pressure, viscosity, and fluid‐structure interaction. In the field of modern medicine, nanofluids are utilized in a major and unique way. Nanofluids, which are suspensions containing various nanoparticles in a base fluid, may exhibit different thermal properties from pure fluids. As an illustration, multiple studies have been carried out using nanofluids, which demonstrate that these fluids′ thermal conductivities are significantly greater. As a result, nanofluids are advantageous for applications involving microscale cooling. Choi and Eastman [2] characterized nanofluids as suspensions of metallic, nonmetallic, carbon‐based, or hybrid particles ranging in size from 1 to 100 nm in base fluids including water, oil, glycol, or refrigerants. Hayat and Nadeem [3] claimed that the silver–copper oxide/water (Ag–CuO/water) hybrid nanofluid enhanced chemical processes, heat generation, and radiation. Selimefendigil and Öztop [4] found that copper (Cu)‐based nanofluids had the highest heat transmission among TiO_2_, Al_2_O_3_, and Cu. Sultan et al. [5] recommend that blood velocity increases and temperature falls with increasing stenosis angle; velocity is unaffected by constant heat flux. Akbar et al. [6] applied wall permeability and thermal buoyancy to model blood flow in stenosed arteries containing Cu nanoparticles. Although Bhatti et al. studied drug transport by nanofluid in porous channels, Sharma et al. [7] investigated magnetic field effects. Das et al. [8] outlined the impact of the hybrid composition of nanoparticles suspended in blood flowing in an inclined stenosed artery under magnetic field orientation. The study of Hussain et al. [9] demonstrates that the amalgamation of Ag and Cu nanoparticles in the bloodstream increases flow velocity, decreases temperature, and enhances chemotherapy in stenosed arteries, thereby improving treatments for vascular diseases through nanofluids. Al‐Kouz et al. [10] asserted that a Carreau nanofluid model, incorporating four distinct types of nanoparticles and solved through spectral relaxation, can accurately forecast the pressure drop and velocity in stenosed arteries, thereby enhancing the detection and treatment of cardiovascular diseases. In stenosed arteries, Thirunanasambantham et al. [11] observed that using COMSOL Multiphysics to simulate hybrid nanofluid blood flow (containing Ag and gold [Au] nanoparticles) under magnetic fields exhibits diminished shear stress, velocity, and poststenotic recirculation, providing encouraging advancements in targeted drug delivery and nanomedicine for cardiovascular therapies. Some research, led by Zaman et al. [12], employs mathematical modeling to examine time‐dependent, non‐Newtonian blood flow incorporating cylindrical Cu and aluminum oxide hybrid nanoparticles within a stenosed artery, utilizing the homotopy investigation method to solve nonlinear equations, suggesting enhanced axial velocity, thermal conductance, and significant potential for magnetic‐targeted nanodrug delivery compared with conventional nanofluids. Tang et al.′s [13] research utilizes numerical solutions using MATLAB′s bvp4c method to explore viscous dissipation in Sisko nanofluid flow with Au nanoparticles in a porous stenosed artery. The outcomes indicate greater velocity with increased volume fraction and curvature, lower temperature with higher Prandtl number (Pr), and implications for atherosclerosis prediction through drag force and heat transfer analysis. To further understand the effects of Brownian motion, thermophoresis, and Grashof numbers, Ellahi et al. [14] theoretically investigate nanofluid blood flow through composite stenosed arteries with permeable walls, clarify nonlinear momentum equations for mild stenosis, answer coupled temperature and nanoparticle equations using the homotopy perturbation method, and obtain exact velocity profile solutions. Flow impedance, pressure gradient, and stream function expressions are graphically evaluated.

Investigation of Cu, TiO_2_, and Al_2_O_3_ nanofluids in a stenotic artery presented that nanoparticles significantly increase axial velocity within the central area and result in a larger trapping bolus compared with pure blood [15]. A semianalytical study of MHD hybrid (Au–Cu) nanofluid flow in a stenotic artery, by means of the Caputo fractional derivative, shows that the fractional order, stenosis height, and flow parameters (Ha, Ra, Ec, Cr, Sc) critically affect the velocity, temperature, and concentration profiles, which are vital for cardiovascular treatments [16]. A numerical study of Ag–Cu nanofluid flow in a stenosed artery showed that enrichment of nanoparticle concentration increases blood velocity but decreases temperature. This achieved its potential use in biomedical applications like chemotherapy [17]. A biomedical engineering study used a Carreau tetra nanofluid mathematical model and the spectral relaxation approach to simulate flow in a stenosed artery. The model effectively forecasts pressure drop and velocity distribution, providing a crucial diagnostic tool for stenosis and cardiovascular diseases [18]. Using the Crank–Nicolson scheme, this work modeled the pulsatile flow of a hybrid (Au–Al_2_O_3_) nanofluid in a porous, stenosed artery under a magnetic field. The hybrid nanoparticles effectively reduce hemodynamic variables and can lead to non‐surgical treatments for atherosclerosis [19]. A novel three‐dimensional (3D) biofluid simulation of blood flow in a narrowing artery with multiple stenoses and multiple thrombi was influenced by hybrid Ag–Au nanoparticles. These findings propose that nanoparticles can act as medication carriers to diminish the flow resistance produced by these blockages [20]. Simulating the flow of a generalized power‐law hybrid (Ag–Au) nanofluid in a stenosed artery using COMSOL Multiphysics, this study found that an external magnetic field combined with the nanofluid significantly reduces shear stress and velocity, improving blood flow for targeted drug delivery [21]. Mehmood et al. [22] investigated the effects of concentric ballooned catheterization and heat transfer on a fractional second‐grade hybrid nano blood flow through an artery with both stenosis and aneurysm. Sarwar et al. [23] observed the importance of Cu nanoparticles in a stenotic artery, indicating that the heat transfer rate rises with nanoparticle concentration. Abdelsalam and Bhatti [24] showed a hybrid nanofluid of nanodiamonds and silica in a catheterized tapered artery under a magnetic field, highlighting the nanoparticles′ hydrophilic traits for biomedicine. Hussain et al. [25] observed hybrid nanoparticles and a magnetic field for drug delivery in a multistenosed tapered artery with an essential thrombus, noting that the magnetic field decreases pressure within the stenosis. Akbar et al. [26] researched the result of various shaped metallic nanoparticles under magnetic force in diverging tapered stenosed arteries to understand their structural application in drug delivery. Sarwar et al. [27] modeled hybrid‐blood nanofluid flow in a cosine‐shaped stenotic artery using similarity transformation to evaluate flow quantities like skin friction and flow rate. Hussain et al. [28] concentrated on Au and Ag hybrid nanoparticles in sidewall split dilated arteries, signifying that the combination of nanoparticles is vital for enhancing thermal conduction. Zar et al. [29] improved an Electro‐magneto‐hydrodynamics (EMHD) model for Cu and graphene hybrid nanofluid in inclined stenotic arteries, displaying that the hybrid nanoparticles decrease blood flow resistance. Dawood et al. [30] presented novel analytical research of nanofluid flow in stenosed arteries, considering a variable pulsatile pressure gradient along with magnetic field and heat transfer effects. Lastly, Shahzad et al. [31] studied a multiply stenosed artery with permeable walls and slip boundary effects, focusing on electro‐osmotic effects and leading an entropy and stability investigation on the flow.

The study by Akbar et al. [32] shows that adding hybrid Cu–titanium oxide nanoparticles and increasing the Hall parameter reduces entropy generation and enhances heat transfer in the peristaltic flow of a nanofluid through a symmetric channel. Experimental results [33] reveal that the velocity of the trihybrid nanofluid declines with curvature and nanoparticle concentration, whereas entropy generation increases with electromagnetic effects; heat transfer is significantly enhanced (about 70% higher than that of blood), with the ANN (artificial neural network)‐BLMT technique demonstrating high predictive accuracy for peristaltic flow dynamics. The investigation by Akbar et al. [34] on kerosene‐alumina nanofluid indicates that aggregated particles diminish irreversibility, that the Bejan number decreases with permeability, and that bolus size decreases as volume fraction increases. Zia et al. [35] studied ANNs based on the Levenberg–Marquardt Backpropagation Scheme (ANNs‐LMBPS) model for EMHD Darcy–Forchheimer flow of UO_2_‐MoS_2_ nanofluid, and the magnetic field reduces velocity while the electric field and permeability increase velocity and heat transfer. Magnetohydrodynamic (MHD) peristaltic flow of Al_2_O_3_‐ZnO nanofluid with Hall current, radiation, and slip is solved numerically to assess velocity, trapping, temperature, heat transfer, and pressure gradient [36]. For MHD Reiner–Philippoff peristaltic flow in a curved channel with Ohmic heating, radial magnetic field, and viscous dissipation, the LMM‐BNN model predicts velocity enhancement near the walls, a decrease at the center, and temperature elevation with increasing Hartmann and Brinkman numbers, and it has 10^−11^ mean‐square errors and is effective [37]. THNFs in PTSCs with MHD increase the temperature, entropy radiation, and heat‐source magnetic number, with heat‐transfer enhancement quantified at 12.11%, whereas the drag force increases in the presence of the magnetic field [38].

Yadav and Singh [39] analyzed how Ag‐TiO_2_ hybrid nanofluid boosts artery blood flow velocity by 41% in irregular stenosis and reduces shear stress relative to overlapping stenosis under electroosmotic MHD effects. Another study [40] investigated the flow of a ternary hybrid nanofluid within a stenosed, aneurysmal artery, integrating microorganisms, MHD, and thermal and chemical effects to evaluate hemodynamics. Also, some studies [41] analyze flow velocity increases with particle fraction and decreases with ZrO2 in the Ag‐GO‐ZrO_2_/blood hybrid nanofluid. The ANN predicts increased wall shear stress in diverging stenosed arteries during EMHD. Some research [42] on Au nanoparticle blood flow shows 13.85% velocity and 5.64% temperature changes in stenosed arteries, with wall shear stress highest in converging and lowest in diverging geometries. The investigation [43] of MHD peristaltic blood flow in flexible coaxial tubes shows lower shear stress than a rigid endoscope; the pressure gradient varies with the inner‐outer wavelength ratio for laparoscopic use. The magnetic field and curvature enhance heat transfer in blood flow through a curved porous artery that contains Brinkman and Darcy layers. This phenomenon has implications for the study of atherosclerosis, aneurysms, clot dynamics, and various vascular diseases [44]. The study [45] of computational fluid dynamics (CFD) analysis of the multistenosed left coronary artery (LCA) utilizing the Carreau, Quemada, and modified Cross models indicates that the Quemada model provides accurate velocity predictions at high Reynolds numbers. In contrast, the Carreau model demonstrates superior performance in predicting wall shear stress. The research [46] of the two‐phase model treats blood as a micropolar fluid in the core and plasma as a Newtonian fluid in the peripheral region of a porous‐layered artery whose wall consists of a Brinkman layer followed by a Darcy layer, and analytical solutions in terms of modified Bessel functions show that the permeabilities of these layers significantly affect the velocity and wall stresses.

The Trihybrid nanofluid exploits a mixture of Ag, Au, and Cu nanoparticles to accomplish superior properties, leveraging their different benefits for both improved thermal performance and potential biomedical applications. Cu nanoparticles are valued for their exceptionally high thermal and electrical conductivity and cost‐effectiveness, serving as the primary component for exploiting the nanofluid′s heat transfer coefficient in cooling systems [47, 48]. Ag nanoparticles are used due to their excellent thermal properties and well‐known broad‐spectrum antimicrobial activity, which is crucial for applications requiring biological stability, such as biomedical strategies or long‐term heat conversion systems where microbial growth is a concern [49, 50]. Au nanoparticles, although contributing to thermal properties, are most appreciated for their chemical inertness and biocompatibility, which helps diminish the corrosive potential of the fluid and prevents aggregation, confirming the nanofluid′s long‐term stability. Furthermore, AuNPs hold unique surface plasmon resonance (SPR) properties, making them ideal for targeted photothermal therapy (PTT) and bioimaging contrast agents in medical contexts [51–53].

Despite the extensive literature on mono‐ and dual‐hybrid nanofluids in stenosed arteries, no study has simultaneously quantified the rheological consequences of trihybrid (Ag–Au–Cu) nanoparticle loading, specifically the transition from parabolic to plug‐like velocity profiles and their effect on poststenotic flow instability and thrombogenic risk across a clinically relevant range of Reynolds numbers and volume fractions. This gap is critical because the same rheological change that enhances thermal conductivity for PTT may exacerbate recirculation‐driven thrombosis. The present study addresses this gap directly.

It is important to distinguish two levels at which the trihybrid novelty operates. At the thermophysical property level, the Brinkman and Maxwell models employed here treat each species′ contribution additively through independent volume fraction terms. The trihybrid novelty is therefore not primarily a transport–property novelty. It is a functional biomedical novelty, which the simultaneous availability of Ag antimicrobial activity, Au‐SPR photothermal targeting, and Cu‐ROS (reactive oxygen species) oxidative stress within a single injectable platform cannot be replicated by any dual‐hybrid combination without sacrificing one of the three therapeutic modalities. The present computational study evaluates the hemodynamic and thermal consequences of deploying this platform at clinically relevant volume fraction values, establishing the rheological cost of the multifunctional loading.

For more of the physiological realism and efficiency in this research, several prospects for future research are presented. The existing formulation of viscosity and thermal conductivity can be adapted to specifically account for more intricate shape‐dependent variables, transcending generalized models to accurately represent the influence of particle geometry on transport phenomena, despite the title emphasizing variable shape. Secondly, future simulations need to move from the existing two‐dimensional domain to a full 3D analysis. This is very important for capturing the complicated, nonaxisymmetric flow patterns that happen in curved and stenosed arterial segments, like the circulation and recirculation zones. This is necessary for recording complicated, nonaxisymmetric flow patterns in curved and stenosed arterial segments, such as circulation and recirculation zones. The shear‐thinning behavior of blood would be more accurately represented by employing more intricate non‐Newtonian constitutive models, such as the Casson models, rather than the anticipated Navier–Stokes incompressible flow model. Also, because magnetic targeting is important in nanomedicine, the model should be improved by adding the effect of an external magnetic field (MHD) to test how well controlled, localized delivery of hybrid nanodrugs to the stenosed area works.

## 2. Mathematical Formulation

**Figure 1:**
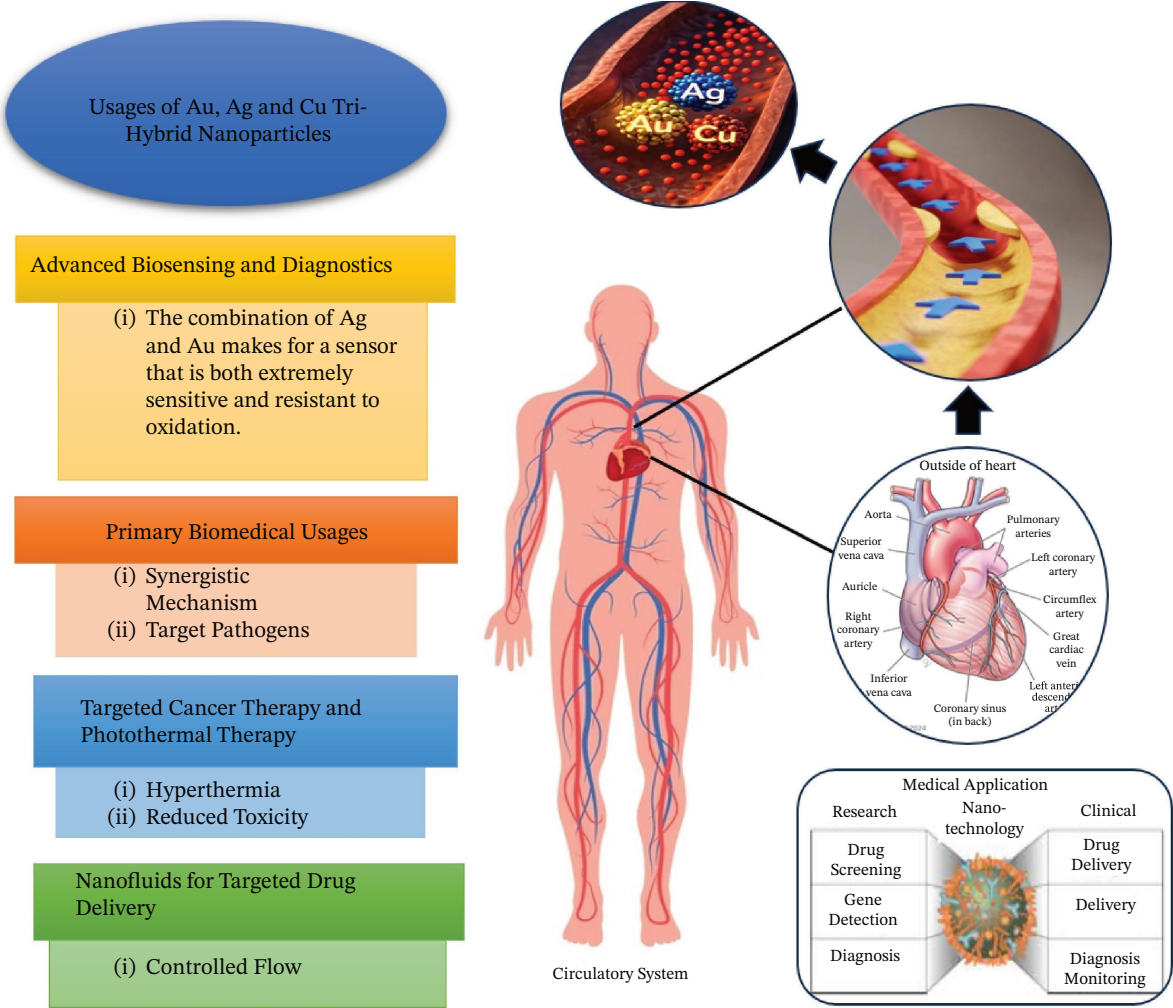
Blood flow with nanoparticles.

Trihybrid nanoparticles (Ag–Au–Cu) are a revolutionary area of nanomedicine, shown in Figure 1, which combines the unique chemical and physical properties of Cu, Au, and Ag into one platform for medicine that works better than any of them alone. Ag is a strong antibacterial agent because it releases ions that can damage the DNA and cell walls of bacteria. Cu, on the other hand, helps make ROS, which stress pathogens by making them go through oxidative stress. Au is a tuner for SPR, which is needed for thermal therapy and imaging tests. It also serves as a stabilizing agent that is safe for living things. In biocompatible usage, one of the main uses of these particles is in PTT. The trimetallic structure in PTT absorbs near infrared light and turns it into heat that kills cancer cells without hurting healthy tissue.

The nanoparticle volume fractions examined in this study, *ϕ*_*T*_ = 3*%*, 9*%*, 15*%*, were selected to span a physically meaningful and computationally instructive range that simultaneously captures biomedically relevant low‐dose conditions and the rheological extremes associated with high nanoparticle loading.

The lower bound of *ϕ*_*T*_ = 3*%*, (*ϕ*_1_ = *ϕ*_2_ = *ϕ*_3_ = 0.01 per species) is consistent with the dilute‐to‐moderate concentration regime widely employed in prior computational studies of nanofluid hemodynamics in stenosed arteries [9, 17, 23], and corresponds to a range where thermophysical property enhancement over pure blood is measurable but viscosity augmentation remains modest. This value also aligns with reported experimental preparation concentrations for metallic nanoparticle suspensions (Ag, Au, and Cu) in aqueous base fluids, where stability can be maintained without significant aggregation [47, 49].

The intermediate value of *ϕ*_*T*_ = 9*%*, (*ϕ*_1_ = *ϕ*_2_ = *ϕ*_3_ = 0.03 per species) was selected to represent a transitional regime in which both viscosity and thermal conductivity are substantially elevated relative to the base fluid, enabling systematic comparison of the competing rheological and thermal effects that are the central focus of this work. Studies of hybrid nanofluid systems in biomedical flow configurations have employed similar intermediate concentrations to identify the onset of plug‐like velocity profiles and enhanced poststenotic recirculation [11, 21].

The upper bound of *ϕ*_*T*_ = 15*%*, (*ϕ*_1_ = *ϕ*_2_ = *ϕ*_3_ = 0.05 per species) was deliberately chosen to represent an upper‐limit therapeutic scenario relevant to localized PTT, wherein high nanoparticle concentrations are introduced directly into the peritumoral vasculature rather than systemically. It is explicitly acknowledged, however, that *ϕ*_*T*_ = 15*%* exceeds currently demonstrated safe systemic intravenous concentrations for metallic nanoparticles in clinical settings. In vivo studies of Au nanoparticle administration report safe systemic concentrations typically at volume fractions of less than 1%, whereas experimental PTT protocols employing localized intratumoral injection have demonstrated therapeutic efficacy at higher local concentrations [51–53]. The *ϕ*_*T*_ = 15*%*, the case is therefore best interpreted as a computational boundary condition that quantifies the maximum rheological and thermal consequences of high nanoparticle loading, providing mechanistic insight relevant to the design and safety optimization of localized delivery protocols, rather than as a directly clinically deployable dose. The equal partitioning of total volume fraction among the three species (*ϕ*_1_ = *ϕ*_2_ = *ϕ*_3_ = *ϕ*_*T*_/3 was adopted for systematic parametric consistency and to isolate the effect of total loading from individual species composition, with unequal partitioning identified as a direction for future investigation.

**Figure 2:**
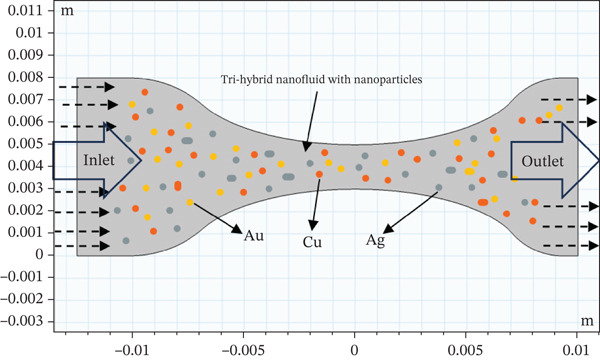
The geometry of an idealized 2D stenotic artery.

The blood was supposed to be an unsteady and incompressible Newtonian fluid moving through a stenotic artery in this research. Here, we employ Ag and Au as nanoparticles and blood as a base fluid. Figure 2 depicts a drawing of the blood flow situation in arterial stenosis, and the coordinate was chosen so that the blood flow is in the desired direction.

The computational domain is a restrained channel, often used to model fluid flow phenomena like blood flow through a stenotic region, and is constructed using sequential Boolean operations. The process starts with a large rectangle with a height of 0.0008 m from which an ellipse with a‐semiaxis 0.00075 m and b‐semiaxis 0.0003 m is subtracted. This subtraction carves out the curved walls, forming the narrow central throat. Finally, to smooth the geometry and ensure a numerically stable boundary for the simulation, a rectangle left 0.0005 m and a rectangle right 0.0003 m radius are applied to round the sharp internal corners of the constricted region and the external corners near the inlet/outlet, before the domain is finalized by the Form Union operation. The “add physics” function was used to incorporate blood flow and heat transport into open‐source software. The entry and departure points were selected, and the velocity at the border was zero. 19.85°C was the standard temperature for heat transmission and laminar flow. With the inlet temperature at the upper boundary being 24.85°C and the outlet temperature at the inferior barrier being 27.35°C, the starting temperature in the heat transfer was 73.15°C. Thermophysical qualities (material properties) of blood were investigated. The time‐dependent model system of equations is then subjected to the mass, velocity, and thermal expansion equations.

## 3. Governing Equations

There appear to be equations in this part that can be tiresome on their own. Yet, we must be familiar with these equations as well as their physical impact because they form the basis of all theoretical and CFD. Because of this, we utilized a lot of time and effort figuring out the governing equations. Considering this time and effort, it is important to now summarize the essential forms of these equations and pause to consider them.

Equation of continuity [54]:

(1) ρ∇·u=0.

Equation of momentum [54]:

(2) ρ∂u∂t+ρu·∇u=∇·−pI+τ+F+ρg.

Equation of energy [55]:

(3) d2ρCp∂T∂t+d2ρCpu·∇T+∇·q=d2Q+q0+d2Qp+d2Qvd.

Here,

(4) Pinit=P+Phydro and Phydro=ρrefg·r−rref.

The boundary conditions for the stenotic artery for Equations (1)–(3) are given below:

For inlet boundary [56]:

(5) u=−uin,u0=Re·μthnfPthnfρthnf·D,u=0.

For outlet boundary:

(6) Phydro=Prefg·r−rref+q,−pI+τ·n=−P0+Phydron,P∧0≤P0,

(7) Pressure⟶static gives P0=0 Pa.

For thermal insulation boundary:

(8) −n·q=0,T=T0.

For heat flux boundary [55]:

(9) n·q=q0,q0=hText−T,

(10) h=Nu·kthnfD,where Nu=4.36 Pipe Laminar Flow,

(11) Pr=μthnf·CPthnfkthnf,

(12) Pe=Re.Pr.

Thermophysical properties of trihybrid nanofluid:

Density of trihybrid nanofluid [57, 58]:

(13) ρthnf=1−ϕ11−ϕ21−ϕ3ρf+ϕ1ρs1+ϕ2ρs2+ϕ3ρs3.

Dynamic viscosity of trihybrid nanofluid [59]:

(14) μthnf=μf1−ϕ12.51−ϕ22.51−ϕ32.5.

Heat capacity of trihybrid nanofluid [58]:

(15) ρCpthnf=1−ϕ11−ϕ21−ϕ3ρCpf+ϕ1ρCps1+ϕ2ρCps2+ϕ3ρCps3.

Thermal conductivity of trihybrid nanofluid (Maxwell model) [60]:

(16) kthnfkf=ks1+22kf−ϕ1kf−ks1ks1+2kf+ϕ1kf−ks1ks2+22kf−ϕ2kf−ks2ks2+2kf+ϕ2kf−ks2ks3+22kf−ϕ3kf−ks3ks3+2kf+ϕ3kf−ks3

The present model treats the trihybrid nanofluid (Ag–Au–Cu/blood) as a single‐phase homogeneous effective medium, wherein the nanoparticle volume fraction *ϕ* is assumed spatially uniform throughout the domain at all time steps. The effective thermophysical properties density (Equation 13), dynamic viscosity (Equation 14), heat capacity (Equation 15), and thermal conductivity (Equation 16) are therefore computed from bulk‐averaged correlations (Brinkman and Maxwell models) using a globally prescribed *ϕ*.

### 3.1. Mesh With Grid Independence Test

**Figure 3:**
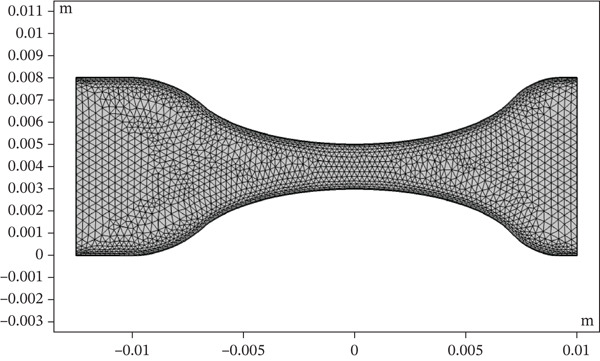
Mesh generation.

In CFD, the mesh is a very important feature. The quality of the mesh affects both the accuracy of the solution and the speed at which it converges. A fine‐element‐size mesh works better and gives more accurate results than a standard‐size mesh. Figure 3 shows typical element size meshes. It was found that the mesh was less refined farther away from the stenosis and more developed in the stenosed area, as shown by the stenosed part.

**Figure 4:**
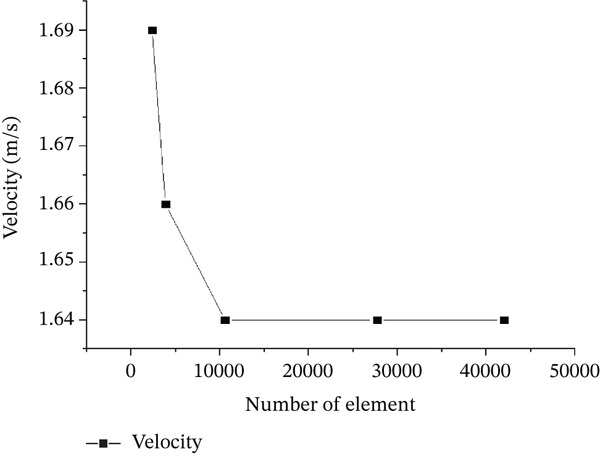
Velocity versus number of elements.

Figure 4 shows the significance of the Mesh Independence Study at *ϕ* = 3*%*, Re = 1200, and *t* = 1.8 s for checking CFD simulations. The simulation results accurately represent the physical scenario and are not merely artifacts of the computational grid resolution.

For geometry discretization, the figure shows how velocity changes with the number of elements used. When the mesh size goes from very coarse to about 10,000, the calculated velocity drops quickly. Therefore, the original meshes did not have the resolution to show how fluids flow properly, especially when there are big changes in speed near the stenosis. After 10,000 elements, the number levels out, and the velocity remains, even if the mesh density improves. The numerical solution has become independent of the mesh due to asymptotic convergence to ensure the accuracy of velocity, temperature, and heat flow for future simulations.

### 3.2. Validation of Code

**Figure 5:**
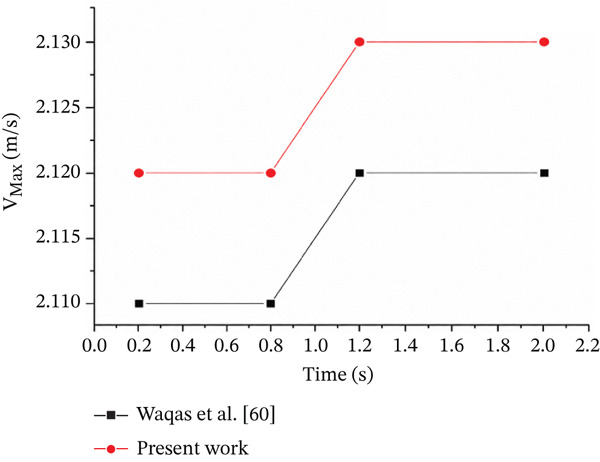
Cross‐validation of V_Max_ against time with existing literature [60].

Figure 5 shows a critical temporal validation of the simulation by comparing the maximum velocity (V_Max_) at the constricted section over time against the work of Waqas et al. [60]. The plot discloses that both the reference work and the present work share the same fundamental flow behavior, V_Max_ remaining nearly constant during the initial acceleration phase, signifying the flow is still developing. Momentously, both data sets undergo a sharp transition and stabilize at their corresponding final velocities at the same moment in time, around *t* = 1.2 s, which characterizes the point where the flow achieves its quasisteady state. Although a minor difference exists in the absolute magnitude, V_Max_ is consistently 0.01 s higher in the present work; the identical stabilization time validates the accuracy of the model′s transient solution and confirms that the selected time points for detailed analysis reliably characterize the stable, fully developed flow regime.

**Figure 6:**
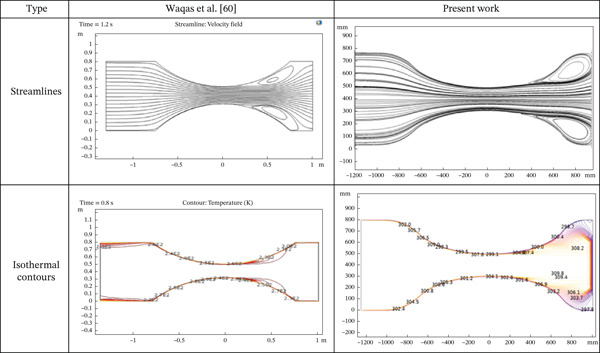
Present work versus Waqas et al.′s [60].

Figure 6 helps as a vital validation benchmark for the entire numerical model by comparing its output with that of Waqas et al. [60]. The top row focuses on the streamline velocity field, where the left panel supports the reference streamlines demonstrating clear flow acceleration at the narrow section and the subsequent formation of large, circulating vortices, or recirculation zones, downstream of the constriction. The present work shows a nearly identical pattern in which the fluid converges and accelerates perfectly, followed by a matching separation and the development of the same large recirculation bubbles, thereby providing strong qualitative confidence that the model accurately maintains the fundamental hydrodynamic behavior of the constricted flow.

The bottom row shows that the model is thermal by comparing isothermal contours at a certain time point. The reference contours show the expected thermal properties. They show that the temperature is very evenly spread out in the bulk fluid core and that the lines get very close together near the walls to show that there is a steep temperature gradient in the thermal boundary layer. The present study accurately replicates this behavior, thereby confirming the model′s solution to the energy equation, as it successfully reflects the uniformity of core temperature and the existence of the expected thermal boundary layers. This double check of both the velocity and temperature fields makes sure that all future results about *ϕ* and Reynolds number effects are based on a strong and dependable computer model.

## 4. Results and Discussion

The computational study examined trihybrid Ag–Au–Cu nanofluid blood flow through a stenosed artery geometry using equal total nanoparticle volume fractions (*ϕ* = 3*%*, 9*%*, 15*%*), Reynolds numbers (Re = 600,1200,1800), and time points (*t* = 0.1, 1, 2 s). The results systematically demonstrate the combined influence of nanoparticle loading and inertial forces on velocity distribution, thermal behavior, shear rate, and pressure fields across all parameter combinations.

The addition of nanoparticles introduces a second, mechanistically distinct influence on this separation process through its effect on the effective viscosity of the nanofluid. Understanding the logical chain connecting nanoparticle loading → viscosity increase → plug‐like velocity profile → modified flow separation is essential for interpreting the parametric trends across *ϕ*_*T*_ = 3*%*, 9*%*, and 15*%* 


**Figure 7:**
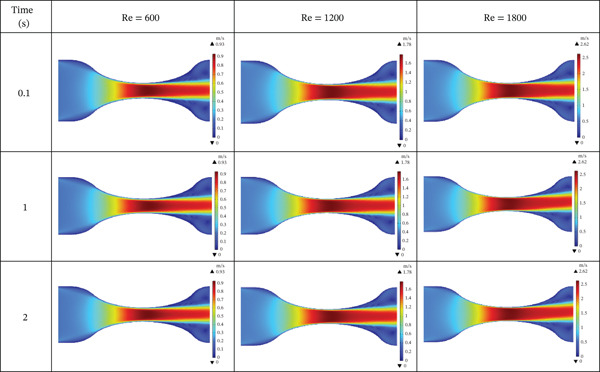
Velocities for trihybrid nanofluid flow at total *ϕ*_*T*_ = 3*%*, **Re** = 600,1200,1800, and *t* = 0.1, 1, 2 s.

**Figure 8:**
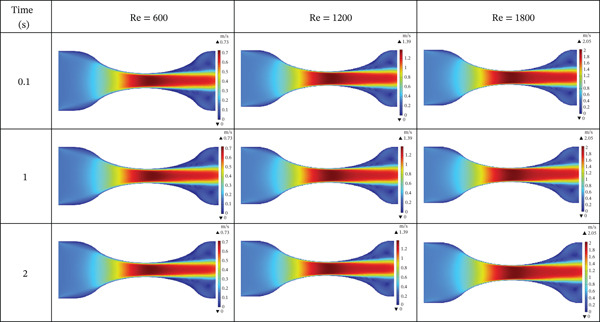
Velocities for trihybrid nanofluid flow at total *ϕ*_*T*_ = 9*%*, **Re** = 600,1200,1800, and *t* = 0.1, 1, 2 s.

**Figure 9:**
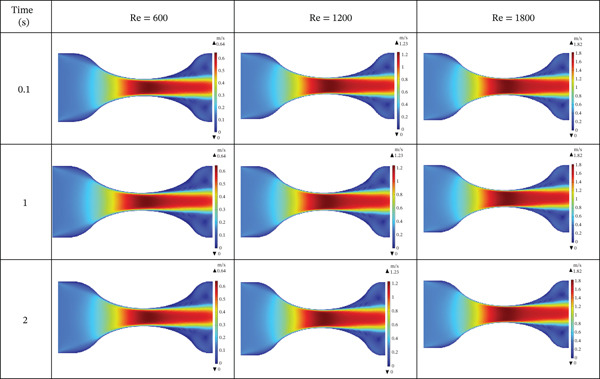
Velocities for trihybrid nanofluid flow at total *ϕ*_*T*_ = 15*%*, **Re** = 600,1200,1800, and *t* = 0.1, 1, 2 s.

**Figure 10:**
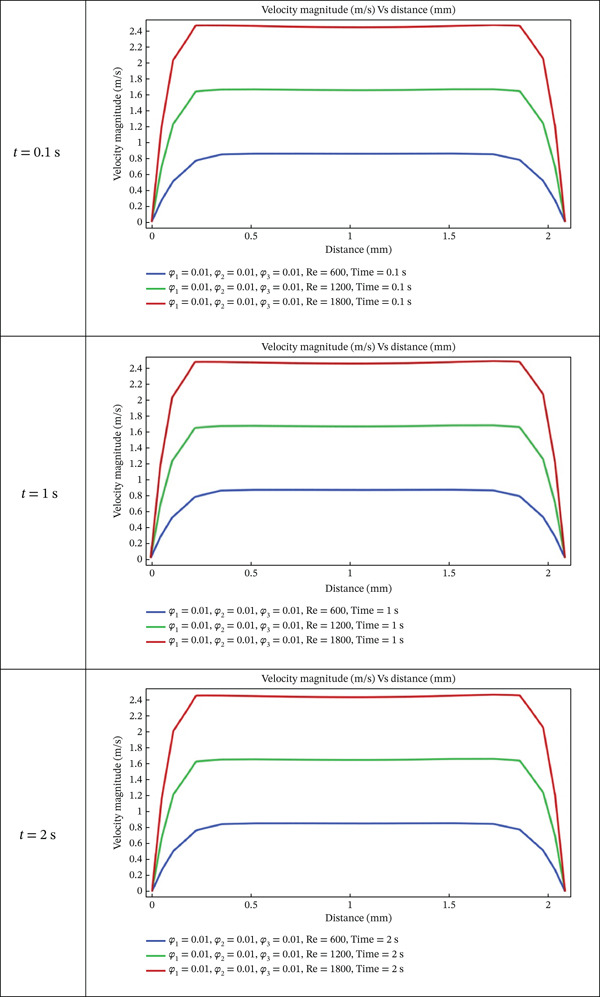
Effects of Reynolds number and total *ϕ*_*T*_ = 3*%* of the velocity in blood (m/s).

**Figure 11:**
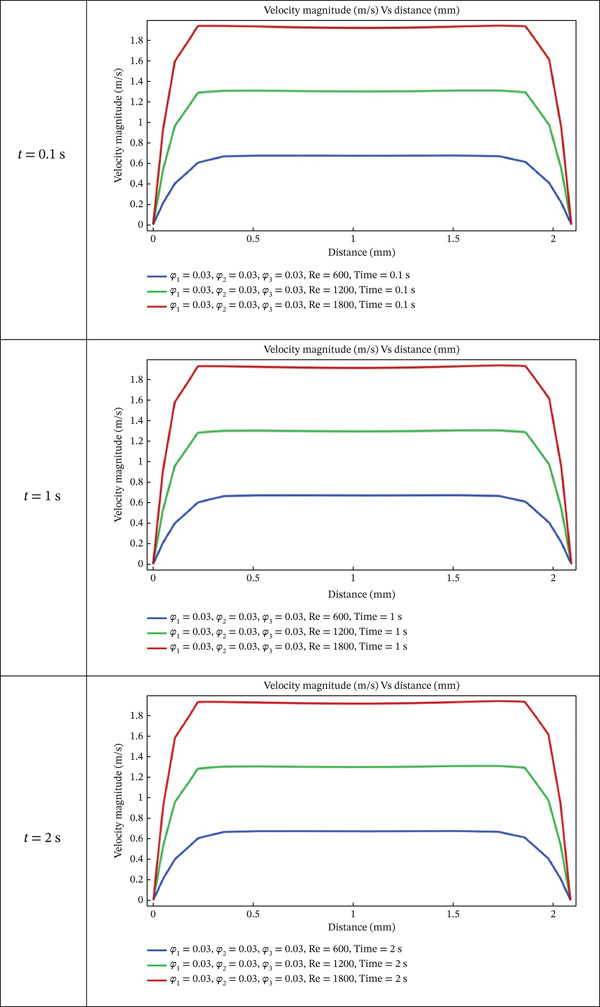
Effects of Reynolds number and total *ϕ*_*T*_ = 9*%* of the velocity in blood (m/s).

**Figure 12:**
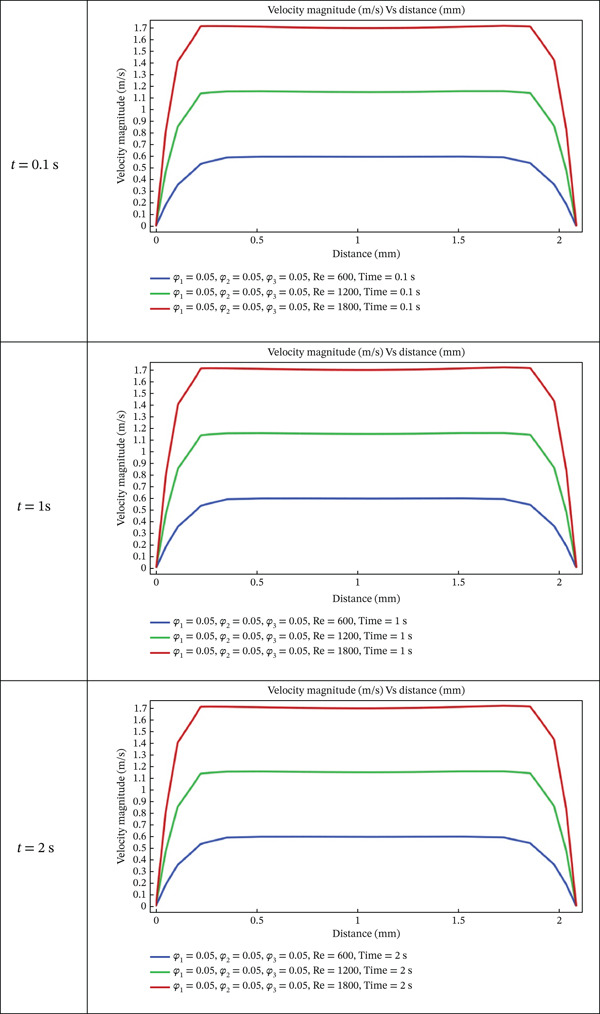
Effects of Reynolds number and total *ϕ*_*T*_ = 15*%* of the velocity in blood (m/s).

As *ϕ*_*T*_ increases, the Brinkman model predicts a nonlinear rise in effective dynamic viscosity at *ϕ*_*T*_ = 15*%* with equal trispecies partitioning, the effective viscosity of the trihybrid nanofluid is higher than that of pure blood. This increased viscosity fundamentally alters how momentum is distributed across the channel cross‐section. In a low‐viscosity fluid, viscous diffusion of momentum from the high‐speed core toward the wall is relatively slow, producing a sharp parabolic velocity profile with a pronounced velocity gradient (high shear rate) near the wall and a peaked core. In a high‐viscosity fluid, momentum diffuses rapidly and uniformly across the cross‐section. The result is that the velocity gradient is progressively compressed into a very thin near‐wall layer, whereas the core velocity becomes nearly uniform; this is precisely the plug‐like, or blunt, velocity profile that is observed with increasing intensity from *ϕ*_*T*_ = 3*%* to *ϕ*_*T*_ = 15*%* in Figure 7, 8, 9, 10, 11, and 12.

This plug‐like flow profile has a paradoxical dual consequence for flow separation at the stenosis exit. On one hand, in the wider prestenotic and poststenotic channel regions, the plug‐like core carries a large mass of fluid at nearly uniform velocity. There is comparatively little momentum variation across the cross‐section to drive flow instability. On the other hand, when this near‐uniform plug of high‐viscosity fluid decelerates upon exiting the constriction into the wider poststenotic region, the deceleration is experienced almost simultaneously across the entire cross‐section rather than being progressive from the wall inward. The thin, high‐shear near‐wall layer, which is the only region retaining a velocity gradient at high *ϕ*_*T*_ is unable to resist the adverse pressure gradient, and flow separation occurs abruptly rather than gradually. Furthermore, the increased viscosity raises the effective inertia of the plug, meaning that once the flow separates and forms a recirculating eddy, the eddy is sustained by a more massive and persistent vortical structure. This explains why the recirculation zones at *ϕ*_*T*_ = 15*%* and Re = 1800 are the largest and most well‐defined of all parametric combinations, despite the absolute velocity being lower than at *ϕ*_*T*_ = 3*%* and the same Re as a result, that is counterintuitive if only velocity magnitude is considered, but physically coherent when the interplay of viscosity, momentum distribution, and adverse pressure gradient is properly accounted for in a certain atmosphere.

### 4.1. Velocity Magnitude Profiles

Figure 7 illustrates the velocity magnitude contours for the trihybrid nanofluid at *ϕ*_*T*_ = 3*%*. In all cases, the velocity is minimum at the inlet and outlet of the wider sections and achieves its peak at the stenosis throat, consistent with mass conservation for an incompressible fluid. At Re = 600, the peak throat velocity is 0.93 m/s, and the velocity contours are smooth and symmetric, with a well‐defined parabolic core profile in the wider sections. At Re = 1200, the peak throat velocity rises to 1.78 m/s; the high‐velocity core (red) at the throat becomes narrower and more concentrated, and a region of very low velocity (dark blue) starts to appear downstream of the stenosis in the recirculation zone. At Re = 1800, the peak velocity reaches 2.62 m/s. The high‐velocity jet at the throat is elongated, and the poststenotic low‐velocity recirculation zone is substantially larger and more pronounced. The contours at *t* = 1 and *t* = 2 s are nearly indistinguishable, confirming quasi‐steady state has been reached by *t* = 1 s at this concentration.

Figure 8 displays the velocity magnitude contours for *ϕ*_*T*_ = 9*%*. By increasing nanoparticle volume fraction, the velocity reduced to 0.73 m/s at Re = 600, 1.39 m/s at Re = 1200, and 2.05 m/s at Re = 1800 compared with *ϕ*_*T*_ = 3*%*. The reduction indicates the increase in the effective dynamic viscosity due to higher nanoparticle loading. The velocity profiles in the wider inlet and outlet sections show a noticeably flatter character compared with *ϕ*_*T*_ = 3*%*. Viscosity is gradually governing the cross‐sectional velocity distribution, especially at Re = 1200 and Re = 1800. The poststenotic low‐velocity recirculation zone exists at Re = 1200 and rises substantially at Re = 1800. Comparing the *t* = 0.1 and *t* = 2 s contours suggests that the flow undergoes a clear temporal evolution at Re = 1800, with the recirculation region expanding and consolidating before achieving a steady configuration by *t* = 2 s.

Figure 9 presents the velocity magnitude contours at the highest concentration *ϕ*_*T*_ = 15*%*. Peak throat velocities are the lowest across all three volume fractions. At Re = 600, the velocity is 0.64 m/s, at Re = 1200 the velocity is 1.23 m/s, and at Re = 1800 the velocity is 1.82 m/s. The most distinctive feature of this case is the highly pronounced plug‐like velocity profile throughout the wider channel sections. The velocity is closely uniform across the entire cross‐section, with the velocity gradient concentrated exclusively in a thin layer near the walls. This behavior is a direct consequence of the high effective viscosity at *ϕ*_*T*_ = 15*%*, which strongly dampens velocity gradients in the core flow. Through the lower absolute velocities, the poststenotic recirculation zone at Re = 1800 is well defined, and at Re = 1200 a distinct separation region becomes visible that was absent at lower concentrations. These observations confirm that increasing *ϕ* simultaneously reduces peak velocity and promotes flow instability downstream of the stenosis due to the abrupt slowing of the viscous plug‐like flow.

The progression from the parabolic profile at *ϕ*_*T*_ = 3*%* to the plug‐like profile at *ϕ*_*T*_ = 15*%* is not merely a visual trend in the contours. It reflects a fundamental change in the mechanism by which the fluid exchanges momentum with the wall. In a parabolic profile, the velocity gradient is distributed across the full channel width, and the wall shear stress is generated by a moderate gradient but acts over a relatively broad near‐wall region. In a plug‐like profile, *∂* *u*/*∂* *y* is concentrated in a thin boundary layer immediately adjacent to the wall; outside this layer, the velocity is nearly uniform, and the fluid behaves as a solid‐body plug. The wall shear stress in this case is generated by a steep gradient in a very thin layer, while the core flow is effectively decoupled from wall friction. This decoupling has two important consequences observed in the present results.

First, the velocity becomes insensitive to wall effects and decreases primarily due to the increased bulk viscosity resisting the inlet momentum flux, explaining the systematic 31% reduction in plateau velocity between *ϕ*_*T*_ = 3*%* and *ϕ*_*T*_ = 15*%* reported in Figures 10, 11, and 12. Second, the plug boundary layer is dynamically fragile, which means that any geometric perturbation, such as the abrupt channel expansion at the stenosis exit, immediately imposes a pressure gradient that this thin layer cannot withstand, triggering the rapid onset of flow separation and recirculation observed at *ϕ*_*T*_ = 15*%* and Re = 1200 condition, at which separation was absent at the same Re for *ϕ*_*T*_ = 3*%*.

### 4.2. Velocity Profiles Versus Distance Line Plots

Figure 10 shows the velocity profiles along the distance of the channel for *ϕ*_*T*_ = 3*%* across three time steps. The profiles show that the velocity is near zero at both inlet and outlet ends of the 2.1 mm domain and grows sharply upon entering the narrow constriction region, before dropping back near the outlet, forming a characteristic top‐hat profile. At *t* = 0.1s, the profiles for Re = 600, 1200, and 1800 yield plateau velocities of approximately 0.73, 1.64, and 2.64 m/s, respectively, in the constricted region. By *t* = 1 and *t* = 2 s, the profiles are nearly identical to the *t* = 0.1 s results, confirming rapid establishment of the quasisteady state. The increasing plateau width with Re demonstrates that higher inertia maintains the accelerated flow over a longer portion of the constriction, while the sharp velocity transitions at both ends of the constriction reflect the geometric constraint of the stenosis.

Figure 11 shows the velocity profiles for *ϕ*_*T*_ = 9*%*. The plateau velocities in the restricted area are lower than in the *ϕ*_*T*_ = 3*%* scenario, with values of 0.64 m/s (Re = 600), 1.28 m/s (Re = 1200), and 1.91 m/s (Re = 1800). The profiles at all three time steps (*t* = 0.1, 1, 2 s) are virtually identical, suggesting that a quasi‐steady state is reached by *t* = 0.1 s at this concentration. Compared with Figure 10, the velocity profiles at *ϕ*_*T*_ = 9*%* shows a slightly broader and flatter plateau region in the constriction, consistent with the more plug‐like flow behavior driven by higher viscosity. The Reynolds number stays the dominant factor controlling absolute velocity magnitude, with Re = 1800 consistently producing the highest velocity across all concentrations and time steps.

Figure 12 presents the velocity profiles for the highest concentration *ϕ*_*T*_ = 15*%*. The plateau velocities in the constricted region are the lowest among all three volume fractions: approximately 0.57 m/s (Re = 600), 1.14 m/s (Re = 1200), and 1.70 m/s (Re = 1800). The profiles are extremely flat throughout the constricted region, with a very sharp transition at the stenosis entry and exit, which is the hallmark of highly viscous plug‐like flow. Comparing Figures 10, 11, and 12, a clear systematic trend emerges: as *ϕ*_*T*_ increases from 3% to 15%, the plateau velocity decreases by approximately 31% at Re = 1800 (from 2.46 to 1.70 m/s) , 30*%* at Re = 1200 (from 1.64 to 1.14 m/s), and 31% at Re = 600 (from 0.83 to 0.57 m/s), demonstrating a consistent and concentration‐dependent attenuation of flow velocity due to increased nanofluid viscosity.

### 4.3. Streamline Profiles

**Figure 13:**
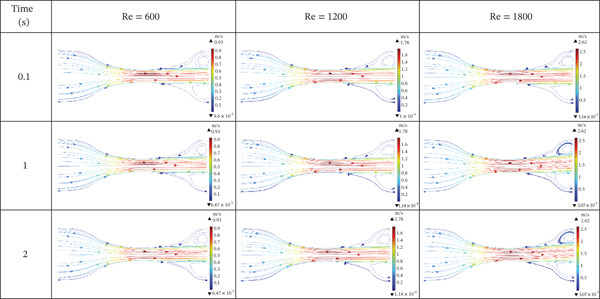
Streamlines for trihybrid nanofluid flow at total *ϕ*_*T*_ = 3*%*, **Re** = 600,1200,1800, and **t** = 0.1, 1,  2 s.

Figure 13 illustrates the streamlines for the trihybrid nanofluid (Au, Ag, Cu) with a volume fraction of *ϕ*_*T*_ = 3*%* (*ϕ*_1_ = *ϕ*_2_ = *ϕ*_3_ = 0.01) at all three Reynolds numbers and steps. At Re = 600, the streamlines are smooth and almost parallel across the whole domain at all times, showing fully laminar and organized flow with a peak velocity of 0.93 m/s at the stenosis throat. There is no recirculation, which demonstrates that viscous forces are stronger than inertial forces at this Re number. At Re = 1200, the velocity is up to 1.78 m/s, and by *t* = 1 s, mild streamline curvature starts to form downstream of the constriction. This indicates that flow separation is starting, with a small recirculation eddy visible in the poststenotic region. At Re = 1800, the highest velocity reaches 2.62 m/s, and a created recirculation zone is visible downstream of the stenosis, growing in size from *t* = 0.1 to *t* = 1 s before stabilizing into a quasisteady state by *t* = 2 s. The streamline patterns at *t* = 1 and *t* = 2 s are nearly identical for Re numbers, indicating that the flow field reaches its quasisteady state by *t* = 1 s, at this concentration.

**Figure 14:**
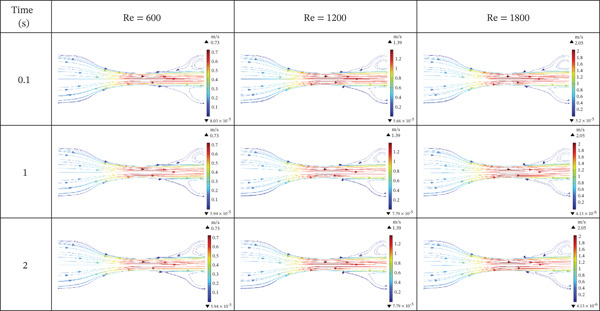
Streamlines for trihybrid nanofluid flow at total *ϕ*_*T*_ = 9*%*, **Re** = 600,1200,1800, and **t** = 0.1, 1,  2 s.

Figure 14 displays the streamlines at a higher volume fraction of *ϕ*_*T*_ = 9*%* (*ϕ*_1_ = *ϕ*_2_ = *ϕ*_3_ = 0.03). Nanofluid effective dynamic viscosity increases with nanoparticle addition. The highest velocities are lower than at the 3% volume fraction. The velocities are 0.73 m/s at Re = 600, 1.39 m/s at Re = 1200, and 2.05 m/s at Re = 1800. At Re = 600, the flow stays fully laminar and smooth at all time steps, with no sign of separation. At Re = 1200, a small but discernible recirculation zone shows downstream of the stenosis by *t* = 1 *s*, more prominent than at the same Re for *ϕ*_*T*_ = 3*%*. At Re = 1800, a recirculation bubble develops downstream of the constriction, and the streamlines exhibit tighter packing near the wall boundaries, showing a more plug‐like core flow. The flow pattern has achieved a stable, closely steady state at all Re by the time *t* = 2 s. A distinct, consistent vortex is present in the recirculation zone at Re = 1800. This suggests that higher viscosity inhibits flow separation at low Reynolds numbers while enhancing instability at high Reynolds numbers.

**Figure 15:**
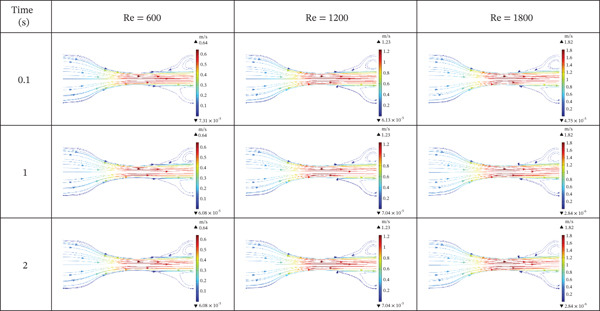
Streamlines for trihybrid nanofluid flow at total *ϕ*_*T*_ = 15*%*, **Re** = 600,1200,1800, and **t** = 0.1, 1, 2 s.

Figure 15 displays the streamlines at a higher volume fraction of *ϕ*_*T*_ = 15*%* (*ϕ*_1_ = *ϕ*_2_ = *ϕ*_3_ = 0.05), where the nanofluid shows the highest viscosity of all three cases. Higher concentrations drop max velocities even further, reaching 0.64 m/s at Re = 600, 1.23 m/s at Re = 1200, and 1.82 m/s at Re = 1800. At Re = 600, the flow stays laminar with no separation at any time step. At Re = 1200, a distinct recirculation eddy shows downstream of the stenosis by *t* = 0.1 s, demonstrating that the combination of high viscosity and moderate inertia develops an earlier onset of flow separation compared with lower concentrations. At Re = 1800, the recirculation zone is the most prominent observed across all cases, with the streamlines showing strong curvature and vortical structures immediately post‐stenosis. The flow achieves quasisteady state by *t* = 1 s and shows no further changes at *t* = 2 s, confirming rapid viscous‐dominated stabilization at this high nanoparticle loading.

### 4.4. Shear Rate Profiles

**Figure 16:**
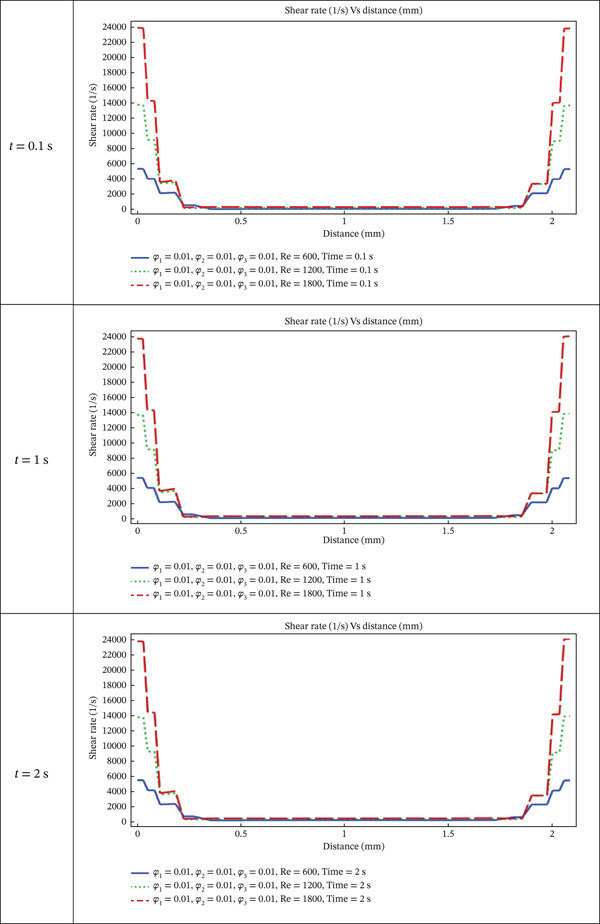
Effects of Reynolds number and total *ϕ*_*T*_ = 3*%* of shear rate in blood (s^−1^).

**Figure 17:**
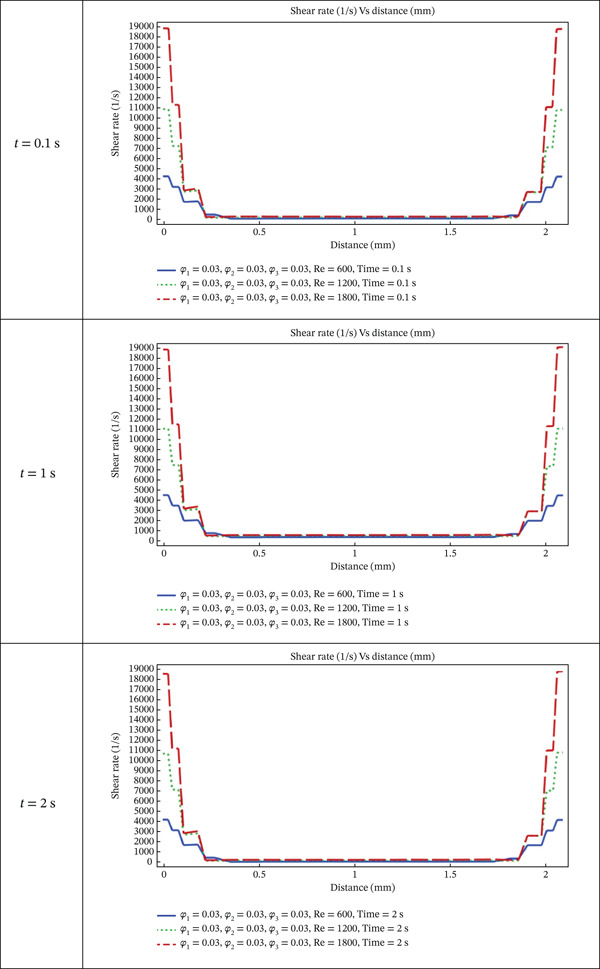
Effects of Reynolds number and total *ϕ*_*T*_ = 9*%* of shear rate in blood (s^−1^).

**Figure 18:**
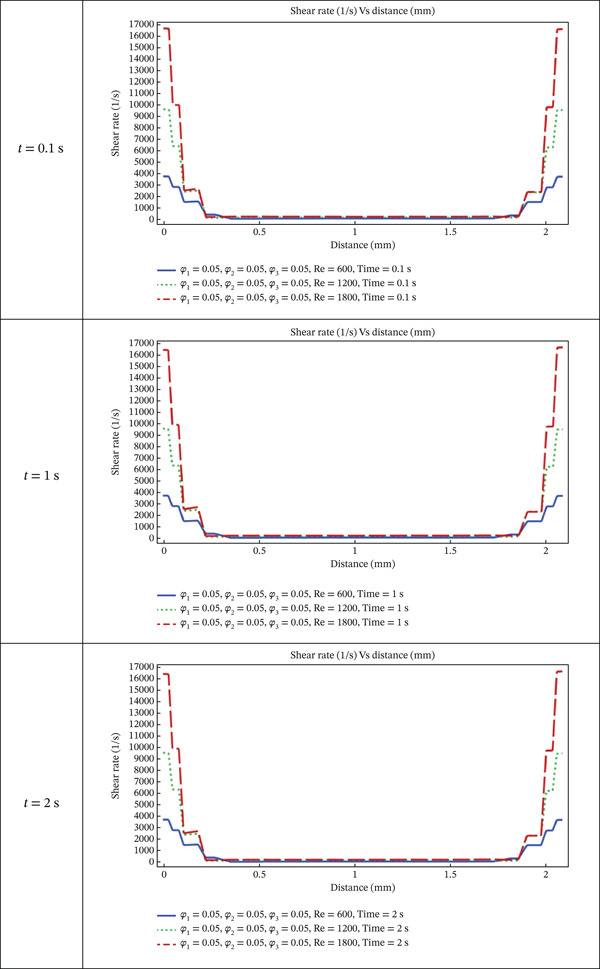
Effects of Reynolds number and total *ϕ*_*T*_ = 15*%* of shear rate in blood (s^−1^).

The shear rate distribution provides direct physical evidence for the velocity profile transitions described above and connects the flow kinematics to the clinically relevant question of platelet activation and thrombosis. Shear rate quantifies the local rate of deformation of the fluid. In a parabolic velocity profile, shear rate is highest at the wall and decreases parabolically toward zero at the channel center line, producing a broadly distributed shear field. In a plug‐like profile, shear rate is concentrated in an extremely thin near‐wall boundary layer and is essentially zero throughout the entire core. The flow shifts from parabolic to plug‐like, so the shear rate becomes very low in the center, but it spikes sharply only where the channel changes shape and the boundary layer adjusts quickly. This is precisely the pattern observed across Figures 16, 17, and 18. The clinical significance of this pattern is that platelet activation is governed by the local shear rate experienced by cells passing through the stenosis boundary layer at the 5000s ^−1^ threshold commonly cited in the thrombosis literature. Because the plug‐like flow at high *ϕ*_*T*_ concentrates all of the shear into a thin layer at the stenosis entry and exit, platelets transiting through these zones experience intense but spatially brief shear exposure. Whether this spatially concentrated, temporally brief high‐shear exposure is more or less thrombogenic than the broader, lower‐magnitude shear distribution of the *ϕ*_*T*_ = 3*%* case depends on the cumulative shear dose, the product of shear rate and exposure time, which cannot be fully evaluated from steady‐state contours alone and represents a direction for future analysis.

Figure 16 presents the shear rate profiles along the arc length for *ϕ*_*T*_ = 3*%*(*ϕ*_1_ = *ϕ*_2_ = *ϕ*_3_ = 0.01). The shear rate profile is characterized by extremely sharp, localized peaks exclusively at the geometric transition zones: near the inlet (0–0.25 mm) and at the outlet (1.85–2.1 mm), where the channel geometry changes abruptly between the wide and narrow sections. In the central constricted region (0.25–1.85 mm), the shear rate is virtually zero, confirming the plug‐like, near‐uniform velocity distribution in the core flow. At *t* = 0.1 s, the peak shear rate at the inlet transition reaches approximately 24,000 s^−1^ for Re = 1800 (red dashed line), approximately 14,000 s^−1^ for Re = 1200 (green dotted), and approximately 5700 s^−1^ for Re = 600 (blue solid). At *t* = 1 and *t* = 2 s, the peak values and spatial distributions are essentially unchanged from *t* = 0.1 s, confirming that the shear rate field also reaches quasi‐steady state rapidly. These high, localized shear rates at the geometric transitions are clinically significant, as they exceed the known platelet activation threshold of approximately 5000 s^−1^ for Re = 1800 and Re = 1200, indicating a risk of mechanically induced thrombosis at the stenosis boundaries.

Figure 17 shows the shear rate profiles for *ϕ*_*T*_ = 9*%*(*ϕ*_1_ = *ϕ*_2_ = *ϕ*_3_ = 0.03).The spatial pattern is identical to Figure 16, with sharp peaks confined to the inlet and outlet transition zones and near‐zero values in the core. However, the peak shear rates are reduced relative to the case: at *t* = 0.1 s, the maximum shear rate at Re = 1800 reaches approximately 19,000 s^−1^, compared with 24,000s^−1^ at *ϕ*_*T*_ = 3*%*.At Re = 1200, the peak is approximately 11,000 s^−1^, and at Re = 600, approximately 4200 s^−1^. The profiles at *t* = 1 and *t* = 2 s are indistinguishable from *t* = 0.1 s, again confirming rapid quasi‐steady state. The reduction in peak shear rate with increasing *ϕ*_*T*_ consistent with the lower velocity gradients produced by the more viscous, plug‐like nanofluid flow. Nevertheless, the peak shear rate at Re = 1800 still substantially exceeds the platelet activation threshold, maintaining clinical concern at high Reynolds numbers even at this elevated concentration.

Figure 18 presents the shear rate profiles at the highest concentration *ϕ*_*T*_ = 15*%*(*ϕ*_1_ = *ϕ*_2_ = *ϕ*_3_ = 0.05). The peak shear rates are the lowest among all three volume fractions: approximately 17,000 s^−1^ at Re = 1800, approximately 9700 s^−1^ at Re = 1200, and approximately 3900 s^−1^ at Re = 600, all at *t* = 0.1 s. At *t* = 1 and *t* = 2 s, the profiles are stable and virtually identical to *t* = 0.1 s. Comparing all three shear rate figures, the trend is clear: increasing *ϕ*_*T*_ from 3% to 15% reduces the peak wall shear rate at Re = 1800 by approximately 29% (from 24000 to 17000 s^−1^). Although this represents a measurable reduction, the shear rate at Re = 1800 still significantly exceeds the platelet activation threshold at all concentrations. Importantly, the core shear rate remains near zero across all cases, confirming the consistent plug‐like flow behavior in the stenosed region regardless of nanoparticle concentration.

The velocity, shear rate, and thermal results presented are derived under the homogeneous effective medium assumption, wherein *ϕ* is treated as spatially uniform. It is instructive, however, to critically examine how physical phenomena neglected by this assumption, specifically, shear‐induced particle migration, nanoparticle aggregation, and margination, could quantitatively and qualitatively modify the reported findings at high volume fractions (*ϕ* = 9*%*–15*%*).

In pressure‐driven flow through a constricted channel, suspended particles experience lateral forces arising from gradients in shear rate across the cross‐section. These forces drive particles from high‐shear near‐wall regions toward the low‐shear channel core, a phenomenon well‐documented in concentrated suspension flows. In the present geometry, the steep shear gradients observed at the stenosis entry and exit transitions (Figures 16, 17, and 18) would, in a two‐phase framework, generate a cross‐sectional *φ* distribution that is enriched at the core and depleted near the walls. Because the Brinkman viscosity model is nonlinearly sensitive to *ϕ*, core enrichment would produce a local effective viscosity in the plug‐like core that is higher than the bulk‐averaged value, whereas the near‐wall viscosity would be correspondingly lower. This would further accentuate the plug‐like velocity profile reported at *ϕ*_*T*_ = 15*%* and could modestly reduce the peak wall shear rates at the geometric transitions relative to the values reported in Figure 16, 17, and 18. Importantly, within the poststenotic recirculation zone, the centrifugal character of the recirculating vortex would tend to sweep particles outward, creating a locally depleted near‐wall *ϕ*_*T*_ in the recirculation region. This suggests that the near‐wall effective viscosity in the recirculation zone may be lower than modeled, implying that the present study could slightly overestimate the stabilizing influence of viscosity on recirculation at high values of *ϕ*_*T*_.

**Figure 19:**
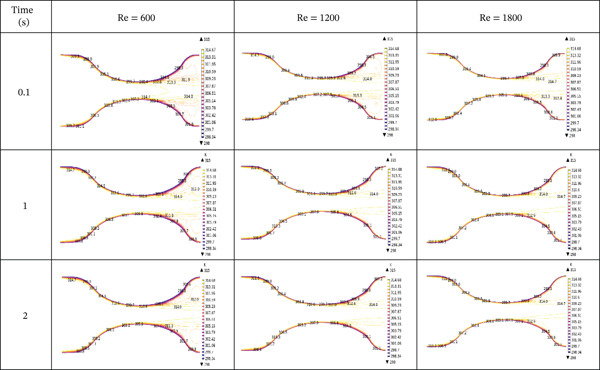
Isothermal contours for trihybrid nanofluid flow at total *ϕ*_*T*_ = 3*%*, **Re** = 600,1200,1800, and *t* = 0.1, 1, 2 s.

**Figure 20:**
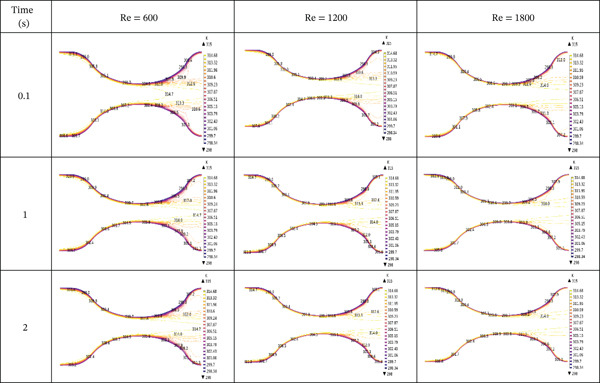
Isothermal contours for trihybrid nanofluid flow at total *ϕ*_*T*_ = 9*%*, **Re** = 600,1200,1800, and *t* = 0.1, 1, 2 s.

**Figure 21:**
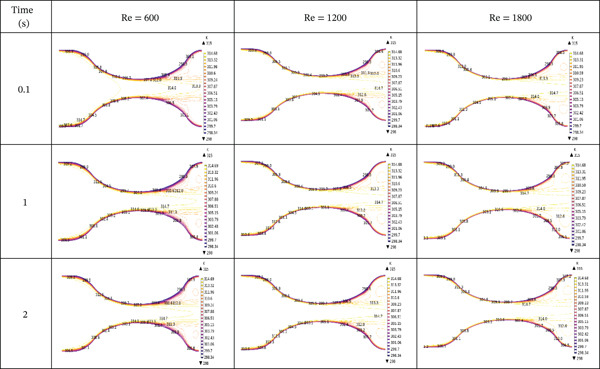
Isothermal contours for trihybrid nanofluid flow at total *ϕ*_*T*_ = 15*%*, **Re** = 600,1200,1800, and *t* = 0.1, 1, 2 s.

At *ϕ*_*T*_ = 15*%*, the interparticle separation distances are sufficiently small that van der Waals and electrostatic interactions among Ag, Au, and Cu nanoparticles may drive the formation of transient aggregates or clusters. Aggregate formation is known to alter both viscosity, in a manner that departs from Brinkman′s single‐particle model, typically increasing viscosity beyond the predicted value for a given bulk *ϕ* and thermal conductivity, where particle chain networks can enhance conductivity beyond Maxwell model predictions by providing preferential heat conduction pathways. The Maxwell model applied here thus provides a conservative lower‐bound estimate of thermal conductivity at *ϕ*_*T*_ = 15*%*, meaning the actual heat transfer enhancement reported in Figures 19, 20, and 21 may be even greater in practice. Conversely, aggregation‐induced viscosity augmentation would reinforce the flow instability and recirculation trends observed at high Reynolds numbers, further strengthening the manuscript′s central finding that high *φ* exacerbates poststenotic flow disruption.

Beyond bulk flow effects, the lateral drift of larger particles or aggregates toward the vessel wall carries direct clinical relevance that the homogeneous model cannot resolve. In tube flow, margination is driven by hydrodynamic interactions between nanoparticles and the near‐wall red blood cell‐depleted plasma layer. Marginated Ag–Au–Cu nanoparticles would accumulate preferentially at the endothelial surface, with two competing consequences. First, near‐wall enrichment of Au nanoparticles would enhance the local SPR effect and PTT efficacy, as the therapeutic agent would be delivered directly adjacent to the target tissue. Second, elevated near‐wall concentrations of Cu nanoparticles, which generate ROS, and Ag nanoparticles, which release cytotoxic ions, could exceed safe endothelial exposure thresholds at *ϕ*_*T*_ = 15*%*, inducing inflammatory injury or endothelial dysfunction. This represents a critical safety consideration for the therapeutic optimization proposed in this work.

### 4.5. Isothermal Contours Profiles

Figure 19 presents the isothermal contours (lines of constant temperature) for *ϕ*_*T*_ = 3*%*. The temperature scale spans from 298 to 315 K across all panels. In all cases, the isothermal lines are concentrated near the inlet and outlet wall boundaries, where the temperature boundary conditions impose the steepest thermal gradients; the central bulk of the domain shows widely spaced isotherms, indicating a relatively uniform temperature field in the core. At Re = 600, the isothermal lines are broadly spread and smooth, with the bulk fluid temperature between approximately 309 and 314 K throughout the domain. At Re = 1200 and Re = 1800, the isotherms near the outlet become progressively more compressed, and the spacing between lines decreases, reflecting a steeper thermal boundary layer driven by increased convective transport. The pattern remains largely unchanged between *t* = 0.1 and *t* = 2 s, confirming early thermal stabilization at this low concentration. The little dependence of the isotherm spacing on Reynolds number confirms that at *ϕ*_*T*_ = 3*%*. The thermal conductivity enhancement is modest, and conduction plays the dominant role in the overall thermal field.

Figure 20 shows the isothermal contours for *ϕ*_*T*_ = 9*%*.Compared with Figure 19, the isotherm lines near the boundaries are more tightly packed, particularly near the outlet and the wider wall sections of the channel. The temperature scale remains between 298 and 315 K. The narrower spacing of isotherms near the walls reflects the enhanced thermal conductivity at *ϕ*_*T*_ = 9*%*, which accelerates heat transfer from the fluid to the wall boundaries and steepens the thermal boundary layer. The bulk fluid core between approximately 305 and 314 K remains relatively uniform. At Re = 1800, the isotherms near the outlet exhibit slight asymmetry compared with the lower Reynolds number cases, indicating some convective distortion of the thermal field. The overall pattern is consistent across all three time steps, confirming that the thermal field stabilizes quickly at this concentration.

Figure 21 presents the isothermal contours for the highest concentration *ϕ*_*T*_ = 15*%*.The isotherm lines are the most tightly clustered near the wall boundaries of all three cases, with very dense packing particularly near the outlet region at all Reynolds numbers. This visual tightening of isotherms directly confirms that the highest nanoparticle loading produces the steepest thermal boundary layers, consistent with the highest effective thermal conductivity among the three concentrations. The bulk fluid core remains thermally uniform between approximately 305 and 314 K, with negligible variation across Reynolds numbers, demonstrating that thermal diffusion at *ϕ*_*T*_ = 15*%* is so effective that the flow velocity has little influence on the core temperature distribution. The isotherm patterns at *t* = 0.1, *t* = 1, and *t* = 2 s are nearly identical, confirming rapid and robust thermal stabilization at this high nanoparticle loading.

The progressive thermal decoupling observed with increasing *ϕ* is quantified by the Péclet number (Pe). At *ϕ*_*T*_ = 3*%*, Pe spans 10,732–32,195 across Re = 600 − 1800, confirming strong advective participation in heat transport. At *ϕ*_*T*_ = 15*%*, the Maxwell‐model enhancement of thermal conductivity reduces the nanofluid Pr to 8.75, yielding Pe = 5124 − 15,372, a 52% reduction relative to *ϕ*_*T*_ = 3*%*, at the same Re. Although all Pe values remain well above unity, indicating that axial advection is never negligible, the substantial reduction in Pr at *ϕ*_*T*_ = 15*%* means that transverse (cross‐sectional) thermal diffusion proceeds fast enough to homogenize the temperature field across the channel width before appreciable axial convection occurs. This is the physical mechanism behind the nearly Reynolds‐number‐independent temperature distribution seen in Figure 21.

### 4.6. Temperature Profiles

**Figure 22:**
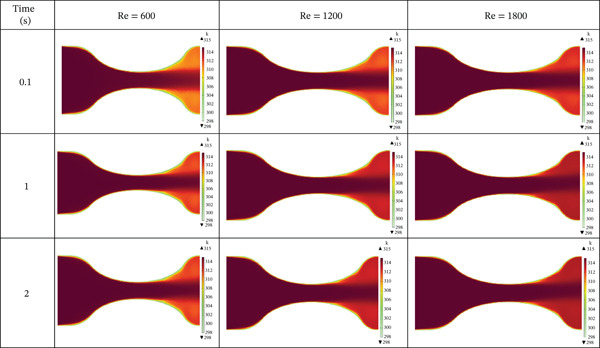
Temperatures for trihybrid nanofluid flow at total *ϕ*_*T*_ = 3*%*, **Re** = 600,1200,1800, and *t* = 0.1, 1, 2 s.

Figure 22 presents the temperature contours for *ϕ*_*T*_ = 3*%*. The color scale spans from 298 (dark, bottom) to 315 K (top). In all panels, the bulk of the fluid domain appears uniformly dark (298–302 K) throughout the wide inlet section, indicating low temperature in the incoming flow. As the fluid enters the stenosis and accelerates, a bright orange‐red region develops along the throat and extends into the downstream section, indicating elevated temperatures between 310 and 315 K driven by viscous dissipation and heat flux from the boundaries. At Re = 600, the high‐temperature region at the outlet wall boundary is narrow and confined. As Re rises to 1200 and 1800, the heated region at the outlet boundary becomes progressively larger and goes further upstream. This is because convective heat transfer is greater at higher flow velocities. The temperature distribution does not change significantly between *t* = 0.1 and *t* = 2 s, suggesting that the thermal field achieves a steady state rapidly.

**Figure 23:**
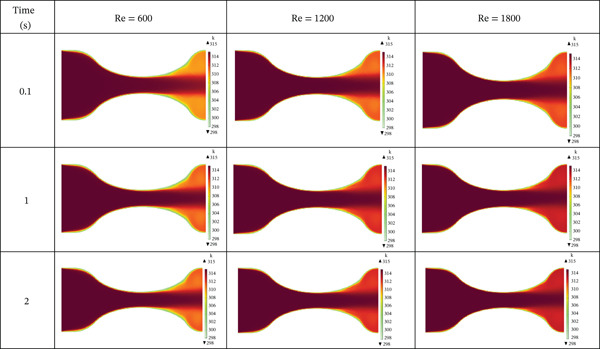
Temperatures for trihybrid nanofluid flow at total *ϕ*_*T*_ = 9*%*, **Re** = 600,1200,1800, and *t* = 0.1, 1, 2 s.

Figure 23 shows the temperature contours for *ϕ*_*T*_ = 9*%*. The overall thermal structure is similar to Figure 22, with a cold inlet region and a warm outlet region. However, the high‐temperature region (between 310 and 315 K) at the outlet boundary is noticeably wider compared with *ϕ*_*T*_ = 3*%*, and the transition from the cold inlet to the warm outlet zone is more gradual. This indicates that the higher thermal conductivity at *ϕ*_*T*_ = 9*%* enables heat to diffuse more effectively through the fluid, producing a broader heated zone. Across all Reynolds numbers, the temperature distribution in the channel is more uniform than at *ϕ*_*T*_ = 3*%*, with the temperature difference between inlet and outlet being maintained consistently at approximately 13 and 17 K. The similarity between the *t* = 0.1, *t* = 1, and *t* = 2 s contours confirms rapid stabilization of the thermal field.

**Figure 24:**
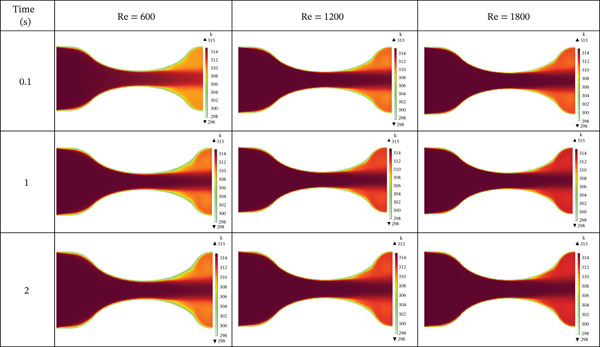
Temperatures for trihybrid nanofluid flow at total *ϕ*_*T*_ = 15*%*, **Re** = 600,1200,1800, and *t* = 0.1, 1, 2 s.

Figure 24 presents the temperature contours for *ϕ*_*T*_ = 15, the case with the highest effective thermal conductivity. The temperature field shows the broadest and most uniform heated region at the outlet boundary among all three concentrations, with the warm zone extending the furthest upstream into the channel at all Reynolds numbers. The overall temperature distribution across the domain is the most homogeneous observed, with relatively smooth transitions from the cold inlet to the warm outlet and minimal Reynolds number dependence. This confirms that at *ϕ*_*T*_ = 15*%*. The thermal conductivity enhancement of the nanofluid is sufficient to dominate the heat transfer process, effectively decoupling it from flow velocity. The contour fields at *t* = 0.1, *t* = 1, and *t* = 2 s are essentially identical, further confirming the thermally dominant character of the highly concentrated nanofluid.

### 4.7. Temperature Profiles Versus Distance

**Figure 25:**
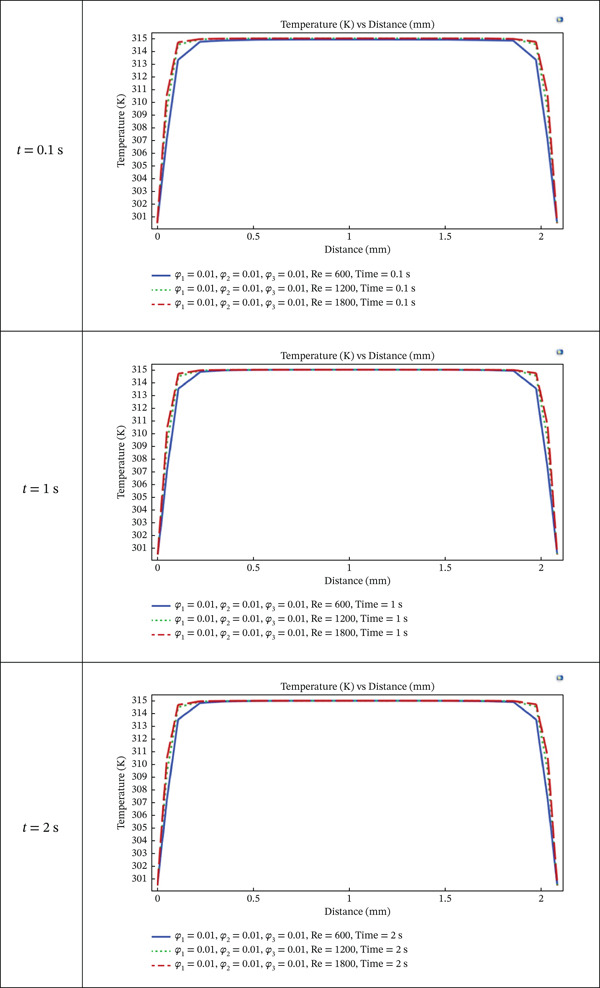
Effects of Reynolds number and total *ϕ*_*T*_ = 3*%* of the temperature in blood (K).

Figure 25 plots the temperature profiles along the distance (0–2.1 mm) for *ϕ*_*T*_ = 3*%* at three time steps. At *t* = 0.1 s, all three Reynolds number curves follow a similar profile: the temperature rises sharply from approximately 301 K at the inlet to a plateau of approximately 315 K between 0.1 and 2.0 mm, then drops sharply back to approximately 301 K at the outlet. The inlet and outlet boundary layers span approximately 0.1–0.2 mm The three Reynolds number curves (Re = 600, 1200, 1800) are closely grouped in the plateau region, with the Re = 600 curve (blue) showing a slightly lower plateau temperature of approximately 314.5 K compared with Re = 1200 and Re = 1800, which both reach approximately 315 K. At *t* = 1 and *t* = 2 s, the profiles are virtually identical to *t* = 0.1 s, confirming rapid thermal steady state. The near‐independence of the plateau temperature from Reynolds number at *ϕ*_*T*_ = 3*%* indicates that conductive heat transfer through the nanofluid dominates over convective effects at this concentration.

**Figure 26:**
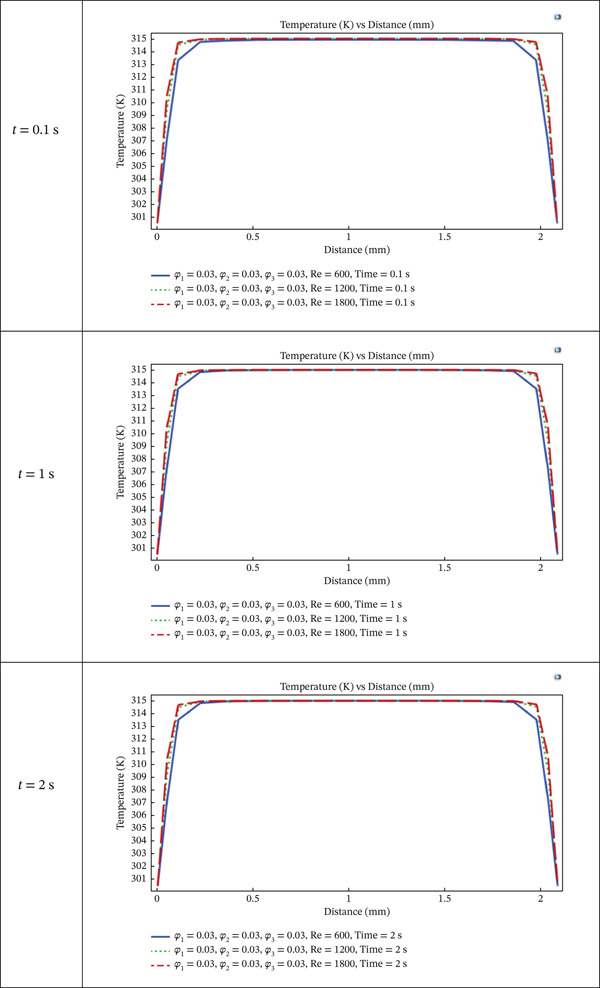
Effects of Reynolds number and total *ϕ*_*T*_ = 9*%* of the temperature in blood (K).

Figure 26 presents the temperature profiles for *ϕ*_*T*_ = 9*%*. The profile shape is very similar to Figure 25, with a sharp rise from approximately 301 K at the inlet to a plateau near 315 K, followed by a sharp drop at the outlet. The key distinction at *ϕ*_*T*_ = 9*%* is that the three Reynolds number curves are even more tightly grouped in the plateau region than at *ϕ*_*T*_ = 3*%*: all three curves converge to a plateau temperature of approximately 315 K with negligible separation between Re = 600, 1200, 1800. The inlet thermal boundary layer appears slightly steeper compared with Figure 25, consistent with the enhanced thermal conductivity of the *ϕ*_*T*_ = 9*%* nanofluid accelerating heat transfer from the wall. At *t* = 1 and *t* = 2 s, the profiles are indistinguishable from *t* = 0.1 s. This near‐perfect overlap across Reynolds numbers confirms that at *ϕ*_*T*_ = 9*%* thermal conductivity enhancement has already begun to make the temperature distribution largely insensitive to flow velocity.

**Figure 27:**
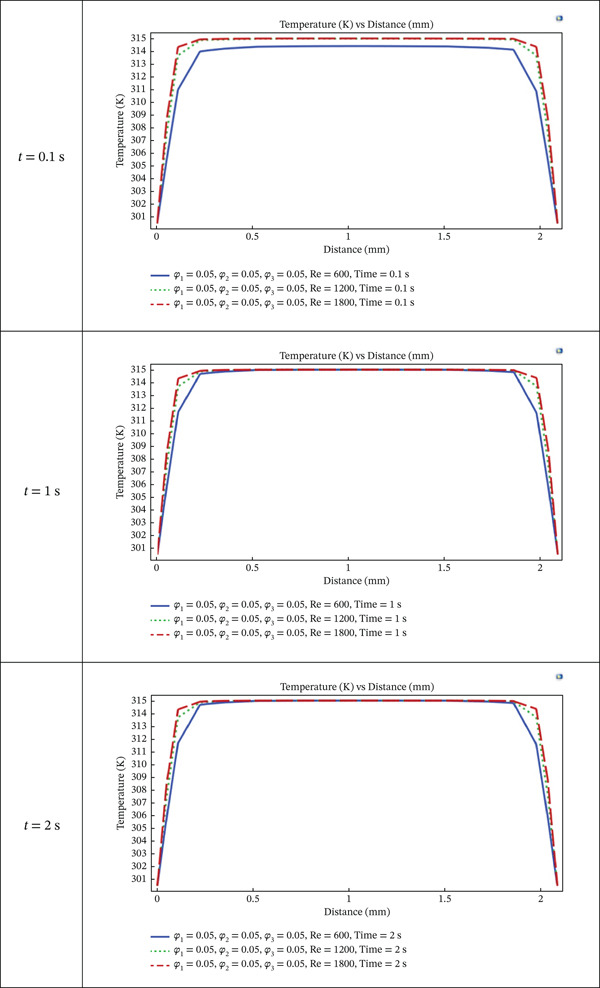
Effects of Reynolds number and total *ϕ*_*T*_ = 15*%* of the temperature in blood (K).

Figure 27 presents the temperature profiles for *ϕ*_*T*_ = 15*%*. The plateau temperature reaches approximately 315 K for Re = 1200 and Re = 1800, whereas the Re = 600 curve (blue solid) plateaus slightly lower at approximately 314.4 K, creating a small but visible separation from the other two curves. This slight separation at Re = 600 is notable. It suggests that at the lowest flow rate, the higher viscosity of the *ϕ*_*T*_ = 15*%* nanofluid reduces advective heat transport sufficiently to create a small but measurable temperature deficit in the core. The inlet boundary layer is the steepest of all three concentrations, confirming the highest thermal conductivity at *ϕ*_*T*_ = 15*%*. At *t* = 1 and *t* = 2 s, the profiles are again stable and identical to *t* = 0.1 s. Overall, comparing Figures 25, 26, and 27, the Reynolds number dependence of the plateau temperature progressively decreases with increasing values of *ϕ*_*T*_, confirming that thermal conductivity enhancement with nanoparticle loading increasingly dominates the heat transfer process over convective effects.

### 4.8. Heat Flux Contours Profile

**Figure 28:**
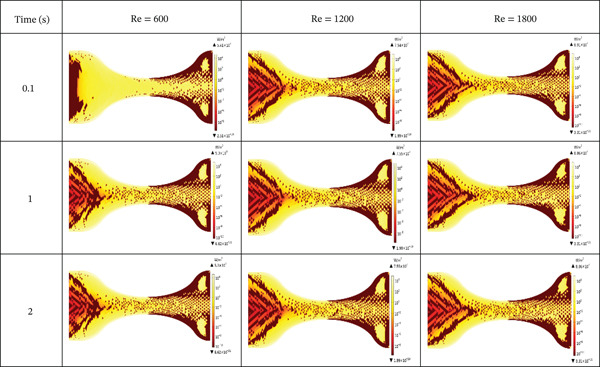
Heat fluxes for Reynolds number at total *ϕ*_*T*_ = 3*%*, **Re** = 600,1200,1800, and *t* = 0.1, 1, 2 s.

Figure 28 displays the heat flux contours, which are plotted on a logarithmic scale for *ϕ*_*T*_ = 3*%*. The maximum heat flux at *t* = 0.1 s is approximately 5.41 × 10^2^W/m^2^ at Re = 600, increasing to approximately 8.91 × 10^2^W/m^2^ at Re = 1800. The highest heat flux is strong at the inlet boundary and circulates as a bright yellow plume enlarging through the narrowed region. The darker maroon regions indicate the downstream and outlet regions, where the heat flux values are lower. At *t* = 0.1 s, the heat flux distribution shows some spatial irregularity at Re = 1800, where the high‐flux plume is asymmetric. By *t* = 2 s, the heat flux distribution stabilizes into a well‐defined steady pattern, with the high‐flux zone consolidating along the inlet boundary and the narrow throat. These results indicate that at *ϕ*_*T*_ = 3*%*, heat exchange is localized primarily at the inlet boundary and the stenosis throat.

**Figure 29:**
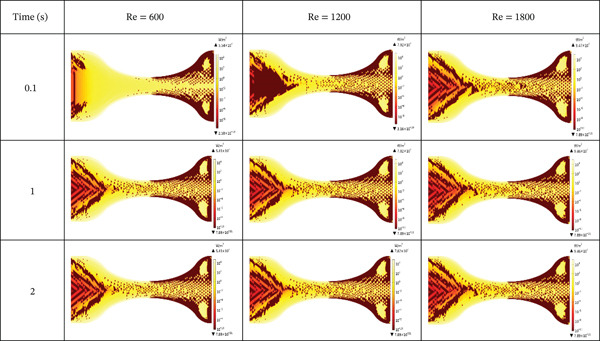
Heat fluxes for Reynolds number at total *ϕ*_*T*_ = 9*%*, **Re** = 600,1200,1800, and *t* = 0.1, 1, 2 s.

Figure 29 illustrates the heat flux contours for *ϕ*_*T*_ = 9*%*. The max heat flux at *t* = 0.1s develops to 5.54 × 10^2^W/m^2^  at Re = 600, showing a little enhancement compared with *ϕ*_*T*_ = 3*%*. At Re = 1800, the highest heat flux achieves 8.91 × 10^2^W/m^2^, heavily consistent with the *ϕ*_*T*_ = 3*%*case. The most significant difference compared with Figure 28 is that the high‐flux yellow region develops over a broader region along the inlet boundary and into the constricted section, indicating the improved thermal conductivity of the *ϕ*_*T*_ = 9*%* nanofluid distributes heat more effectively over a larger surface region. By *t* = 2 s(max  5.41 × 10^2^W/m^2^), the distribution achieves a stable quasisteady pattern that is more spatially uniform along the boundaries than the *ϕ*_*T*_ = 3*%* case, suggesting that higher nanoparticle loading promotes more consistent boundary heat exchange.

**Figure 30:**
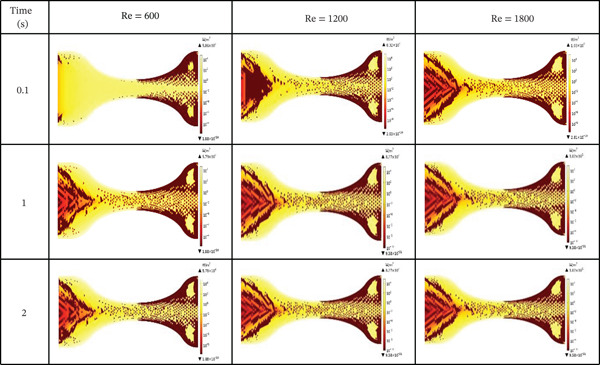
Heat fluxes for Reynolds number at total *ϕ*_*T*_ = 15*%*, **Re** = 600,1200,1800, and *t* = 0.1, 1, 2 s.

Figure 30 illustrates the heat flux contours for *ϕ*_*T*_ = 15*%*, which shows the highest heat flux values among all three concentrations: 5.86 × 10^2^W/m^2^ at Re = 600 and increasing to higher values at Re = 1800. The high‐flux region (bright yellow) is the most extensive of all three cases, circulating broadly along the entire inlet boundary and extending well into the narrow throat section. This indicates that the highest nanoparticle concentration creates the most effective and broadly distributed heat transfer across the fluid‐wall interface. The dark maroon regions in the downstream and outlet sections are smaller than those with lower concentrations. This suggests that the higher thermal conductivity at *ϕ*_*T*_ = 15*%* minimizes the low‐flux dead zones in the poststenotic region. The heat flux distribution achieves quasisteady state by *t* = 1 s and shows negligible further change at *t* = 2 s.

### 4.9. Heat Flux Profiles

**Figure 31:**
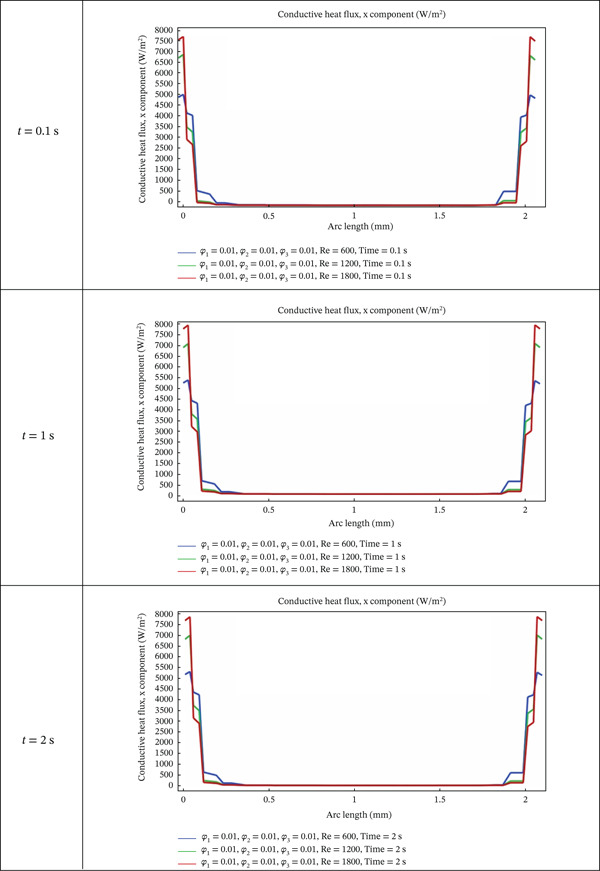
Effects of Reynolds number and total *ϕ*_*T*_ = 3*%* of heat flux in blood (W/m^2^).

Figure 31 plots the *x*−component of the conductive heat flux along the distance (0–2.1 mm) for *ϕ*_*T*_ = 3*%*. The profiles are dominated by two sharp positive peaks located at the inlet and outlet transition zones, and a region of near‐zero conductive heat flux in the central constricted section. At *t* = 0.1 s, the peak heat flux at the inlet achieves 7,600 W/m^2^ for Re = 1800, 6,900 W/m^2^ for Re = 1200 (green), and approximately 5,100 W/m^2^ for Re = 600 (blue). Correspondingly, the outlet peak values mirror the inlet peaks. Between *t* = 0.1 and *t* = 1 s, the profiles are virtually indistinguishable, suggesting that the conductive heat flux field stabilizes almost instantly. The near‐zero values in the core constricted region confirm that conductive heat transport in the flow direction is negligible in the central stenosed section, and all significant conductive heat exchange occurs at the geometric transitions.

**Figure 32:**
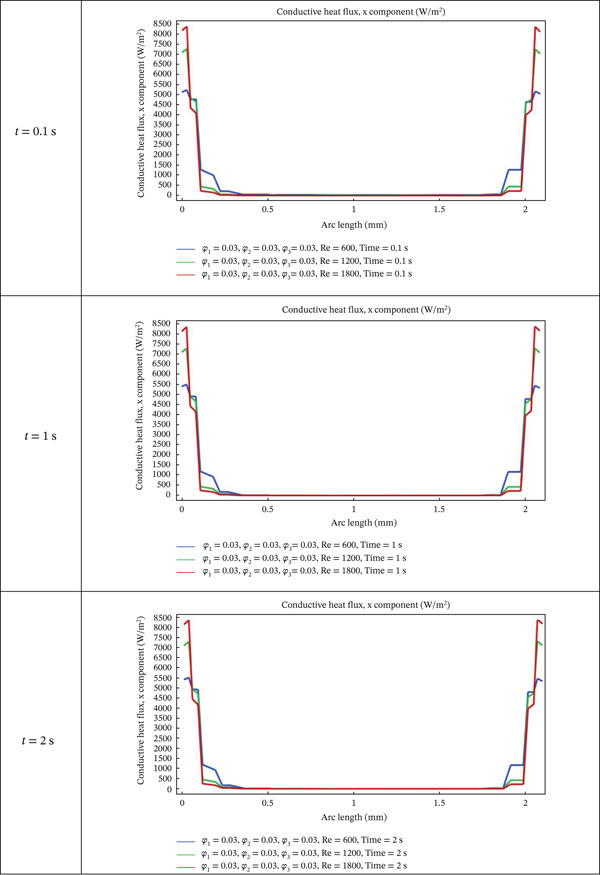
Effects of Reynolds number and total *ϕ*_*T*_ = 9*%* of heat flux in blood (W/m^2^).

Figure 32 illustrates the conductive heat flux profiles for *ϕ*_*T*_ = 9*%*. The overall profile shape is the same as Figure 31, with sharp inlet and outlet peaks and near‐zero values in the central section. The peak heat flux values at the inlet and outlet are notably higher than at *ϕ*_*T*_ = 3*%*: 8,200 W/m^2^ at Re = 1800, approximately 7,100 W/m^2^ at Re = 1200, and approximately 5,200 W/m^2^ at Re = 600, all at *t* = 0.1 s. This increase in peak conductive heat flux directly reflects the higher thermal conductivity of the *ϕ*_*T*_ = 9*%* nanofluid, which drives a more intense conductive heat exchange at the boundary transition zones. A notable feature compared with *ϕ*_*T*_ = 3*%* is that the three Reynolds number curves are more closely spaced at *ϕ*_*T*_ = 9*%*, particularly in the steady‐state plots at *t* = 1 and *t* = 2 s, confirming that the enhanced thermal conductivity reduces the sensitivity of the heat flux profile to flow velocity at this concentration.

**Figure 33:**
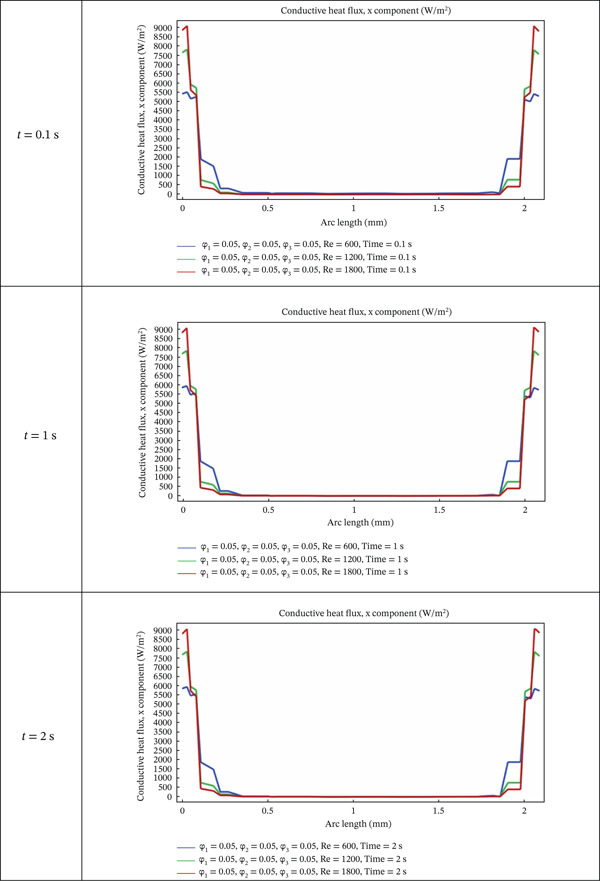
Effects of Reynolds number and total *ϕ*_*T*_ = 15*%* of heat flux in blood (W/m^2^).

Figure 33 presents the conductive heat flux profiles for *ϕ*_*T*_ = 15*%*, which shows the highest peak values of all three concentrations. At *t* = 0.1 s, the inlet peak heat flux achieves 9000 W/m^2^ for Re = 1800, approximately 7900 W/m^2^ for Re = 1200, and approximately 5700 W/m^2^ for Re = 600. The outlet peaks are similarly elevated. Compared with *ϕ*_*T*_ = 3*%* and 9*%*, the profiles at *ϕ*_*T*_ = 15*%* show a wider spatial extent of the elevated heat flux near the inlet, with the peak region spanning a broader arc length range before dropping to near zero. At *t* = 1 and *t* = 2 s, the profiles are stable and identical, and the Re = 1200 and Re = 1800 curves converge more closely than at lower concentrations, with nearly overlapping peaks. The Re = 1200 and Re = 1800 curves at quasisteady state indicate that at *ϕ*_*T*_ = 15*%*, the conductive heat flux in the flow direction has become mostly insensitive to the flow Reynolds number, with thermal conductivity controlling over convective transport in governing the rate and spatial distribution of heat exchange.

### 4.10. Pressure Profiles

**Figure 34:**
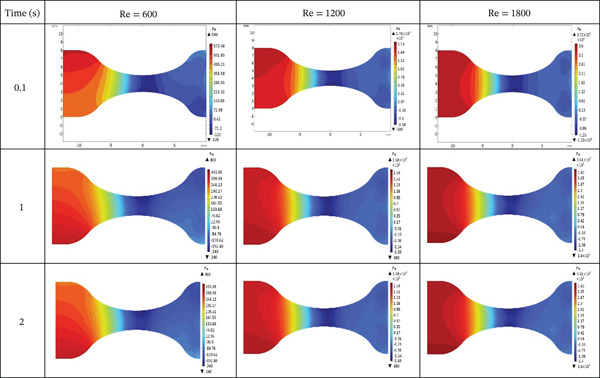
Pressures for trihybrid nanofluid flow at total *ϕ*_*T*_ = 3*%*, **Re** = 600,1200,1800, and *t* = 0.1, 1, 2 s.

Figure 34 presents the gauge pressure contours for *ϕ*_*T*_ = 3*%*. At Re = 600, the pressure field is smooth and gradually transitions from a modest positive pressure of approximately 480 Pa at the inlet to near zero at the outlet, with a gentle pressure gradient reflecting viscous‐dominated flow at low inertia. At Re = 1200, the inlet gauge pressure achieves 1.68 × 10^3^ Pa, and the pressure drop across the stenosis is clearly visible as a sharp color gradient through the narrow throat; the downstream region shows a slight pressure recovery. At Re = 1800, the inlet gauge pressure rises to 3.52 × 10^3^ Pa, and the pressure contours show a steep gradient through the constriction with a low‐pressure zone immediately downstream associated with the recirculation region. The *t* = 1 and *t* = 2 s contours are indistinguishable from *t* = 0.1 s, confirming an early steady state in the pressure field.

**Figure 35:**
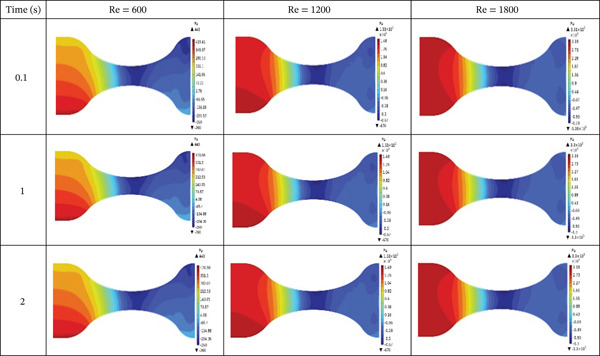
Pressures for trihybrid nanofluid flow at total *ϕ*_*T*_ = 9*%*, **Re** = 600,1200,1800, and *t* = 0.1, 1, 2 s.

Figure 35 illustrates the gauge pressure contours for *ϕ*_*T*_ = 9*%*. The inlet pressure is 440 Pa at Re = 600, which is lower than at *ϕ*_*T*_ = 3*%*. This shows that the flow speed is slower at the same Reynolds number because the viscosity is higher. At Re = 1200, the inlet pressure is 1.31 × 10^3^ Pa, and at Re = 1800 it achieves 3.31 × 10^3^ Pa. The pressure contours at *ϕ*_*T*_ = 9*%* have larger and more uniformly scaled color changes compared with those at *ϕ*_*T*_ = 3*%*. This indicates that the higher viscosity makes the pressure gradient through the stenosis less sharp. A mild low‐pressure region is visible downstream of the constriction at Re = 1800, associated with the recirculation zone visible in the streamline plots. The *t* = 1 and *t* = 2 s pressure fields are stable and consistent with *t* = 0.1 s.

**Figure 36:**
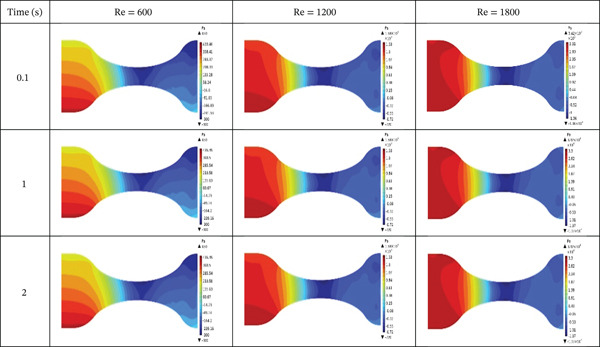
Pressures for trihybrid nanofluid flow at total *ϕ*_*T*_ = 15*%*, **Re** = 600,1200,1800, and *t* = 0.1, 1, 2 s.

Figure 36 presents the gauge pressure contours for the highest concentration *ϕ*_*T*_ = 15*%*. At Re = 600, the inlet pressure is approximately 450 Pa. At Re = 1200, the inlet pressure is 1.31 × 10^3^ Pa, broadly similar to the *ϕ*_*T*_ = 9*%* case. At Re = 1800, the peak inlet pressure is 3.42 × 10^3^ Pa, slightly higher than the *ϕ*_*T*_ = 9*%* result, which is counterintuitive but consistent with the increased viscous resistance offered by the highly concentrated nanofluid requiring a greater driving pressure to maintain the same Reynolds number. The pressure gradients through the stenosis are the most diffuse of all three concentrations, with very smooth and broadly graded color transitions, confirming maximum viscous buffering of the pressure field at *ϕ*_*T*_ = 15*%*. The steady‐state pressure fields at *t* = 1 and *t* = 2 s are stable and match *t* = 0.1 s.

### 4.11. Pressure Versus Distance

**Figure 37:**
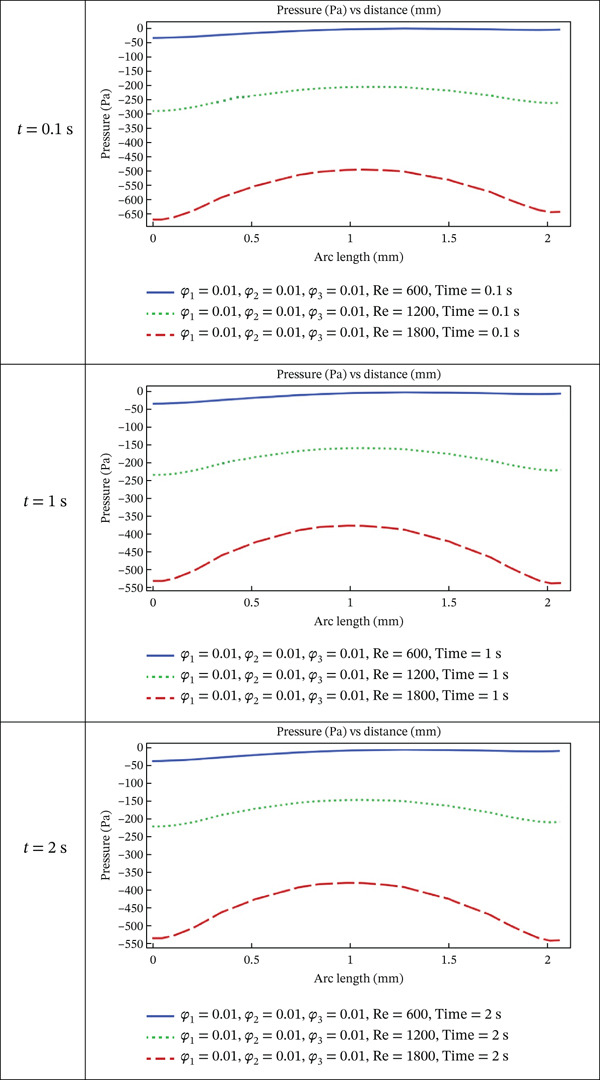
Effects of Reynolds number and *ϕ* = 3*%* of pressure in blood (Pa).

Figure 37 plots the pressure profiles along the arc length (0–2.1 mm) for *ϕ*_*T*_ = 3*%* across three time steps. All pressures are negative relative to the outlet reference, indicating that the outlet is set as the reference zero. At *t* = 0.1 s, Re = 600 shows a nearly flat pressure profile ranging from approximately −25 Pa at the inlet to near 0 Pa at the outlet. As a result, the shape of the stenosis Re = 1200 drops from approximately −280 Pa at the inlet to −230 Pa, before recovering toward 0 Pa at the outlet, exhibiting a characteristic mid‐arc pressure recovery. Re = 1800 shows the steepest profile, dropping from approximately −650 Pa at the inlet to a minimum of approximately −420 Pa mid‐arc and recovering to approximately −520 Pa at the outlet. At *t* = 1 and *t* = 2 s, the profiles are nearly identical to *t* = 0.1 s with only minor flattening of the Re = 1800 curve, confirming quasisteady state. The nonmonotonic pressure recovery mid‐arc, particularly pronounced at Re = 1800, is a signature of the recirculation‐induced pressure redistribution downstream of the stenosis.

**Figure 38:**
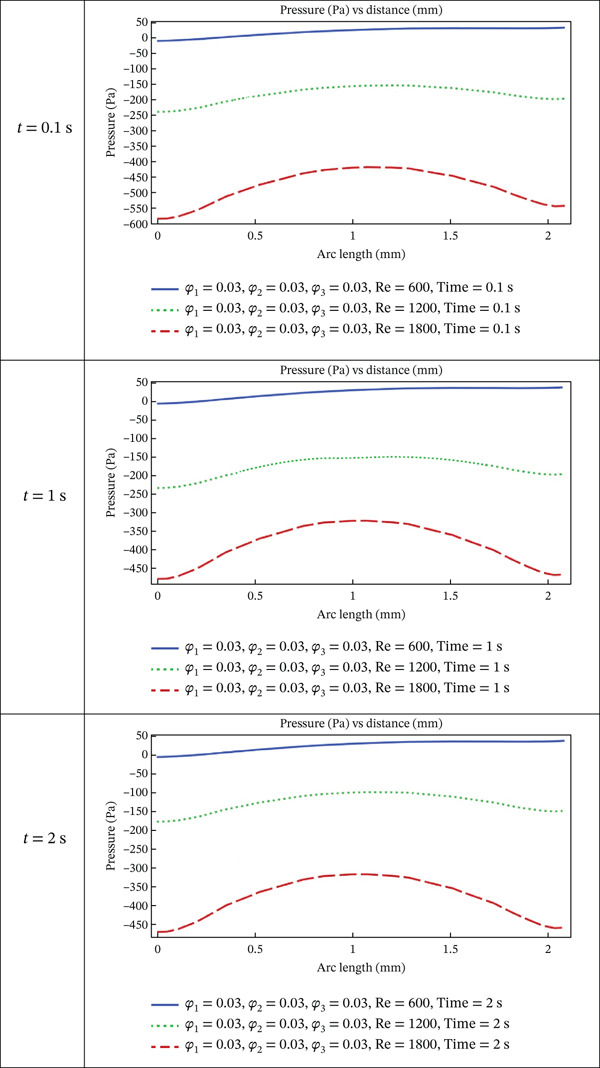
Effects of Reynolds number and *ϕ* = 9*%* of pressure in blood (Pa).

Figure 38 shows the pressure profiles for *ϕ*_*T*_ = 9*%*. The profile shapes are similar to Figure 37 but with notable differences in magnitude and slope. At *t* = 0.1 s, Re = 600 is nearly flat and close to 0 Pa throughout the domain, significantly flatter than the *ϕ*_*T*_ = 3*%* case. Re = 1200 shows a pressure range of approximately −230 Pa at the inlet to approximately −170 Pa at the outlet, a noticeably reduced pressure difference compared with *ϕ*_*T*_ = 3*%*, Re = 1800 drops from a −600 Pa at the inlet to a minimum near −310 Pa mid‐arc, representing a shallower curve than at *ϕ*_*T*_ = 3*%*. At *t* = 1 s, the Re = 600 curve shifts to positive values, indicating a slight positive pressure build‐up due to the altered viscous flow at this concentration. At *t* = 2 s, the profiles are stable and virtually identical to *t* = 1 s. The overall reduction in pressure gradient magnitude at *ϕ*_*T*_ = 9*%* compared with *ϕ*_*T*_ = 3*%* confirms that the higher nanofluid viscosity partially attenuates the inertia‐driven pressure losses through the stenosis.

**Figure 39:**
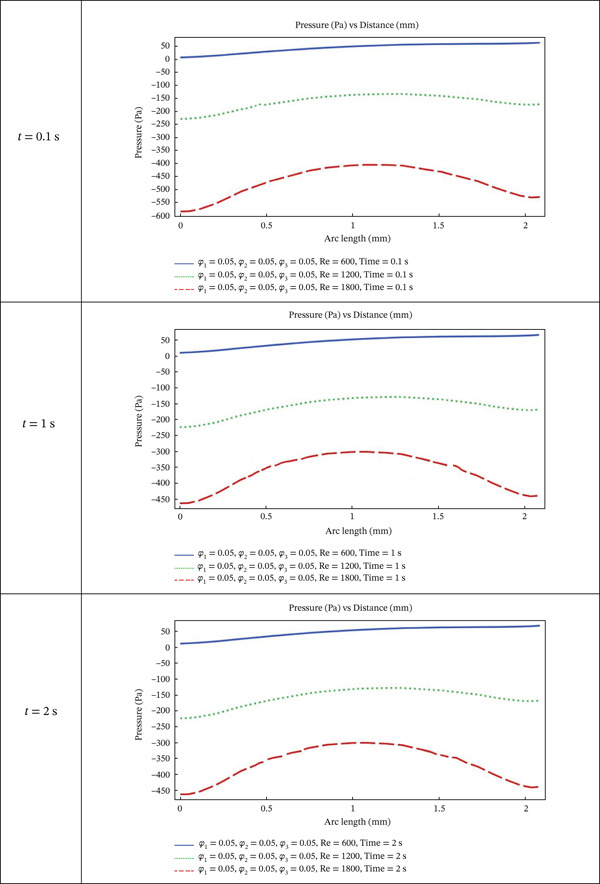
Effects of Reynolds number and *ϕ* = 15*%* of pressure in blood (Pa).

Figure 39 presents the pressure profiles for the highest concentration *ϕ*_*T*_ = 15*%*. At *t* = 0.1 s, the Re = 600 curve is positive across the entire domain, rising gently from approximately 10 Pa at the inlet to approximately 60 Pa at the outlet, indicating a slight net positive pressure gradient at this concentration and Reynolds number. Re = 1200 ranges from approximately −220 Pa at the inlet to approximately −170 Pa at the outlet, broadly similar to the *ϕ*_*T*_ = 9*%* case. Re = 1800 shows the largest magnitude, ranging from approximately −460 Pa at the inlet to a minimum of approximately −310 Pa mid‐arc and recovering to approximately −370 Pa at the outlet. Compared with *ϕ*_*T*_ = 3*%* (Figure 37), the *ϕ*_*T*_ = 15*%* pressure profiles at Re = 1800 show a reduction in peak inlet pressure magnitude from approximately −650 Pa to approximately −460 Pa, a 29% reduction attributable to the viscous buffering effect of the highly concentrated nanofluid. The profiles at *t* = 1 and *t* = 2 s are stable and nearly identical to *t* = 0.1 s, confirming rapid pressure field stabilization at this concentration. In the stenosed artery model, Figures 37, 38, and 39 show that as *ϕ*_*T*_ goes up, there is a clear and regular decrease in inertia‐driven pressure gradients. This indicates that nanoparticle‐enhanced viscosity is a key factor in managing the flow of blood and the pressure in the body.

Thermophysical properties in Table 1 demonstrate nanoparticles (Ag, Au, Cu) superior density (up to 19,300 kg/m^3^ for Au), thermal conductivity (up to 429 W/mK for Ag), and heat capacity variations, qualifying trihybrid nanofluids to improve blood′s heat transfer and viscosity, encouraging recirculation for drug delivery but risking pressure drops and thrombosis at high *ϕ* (such as 15%).

**Table 1:** Description of thermophysical characteristics of the base fluid and nanoparticles.

Property	Density (kg/m^2^)	Heat capacity (J/kgK)	Thermal conductivity (W/mK)	Dynamic viscosity (Pa/s)
Blood	1063	3,746	0.52	0.003
Silver	10,500	235	429	0.005
Gold	19,300	129	310	0.00464
Copper	8933	385	398	0.004

Table 2 indicates that the average element quality of 0.7879 out of 1.0 is good enough, and that the elements are well‐shaped. The mesh consists of 3856 total elements, covering a domain area of 107.1 mm². There are a few elements with a minimum quality of 0.2282, most likely near the sharp edges of the stenosis throat, which is a known geometric problem. This table indicates that the mesh is small enough to see the sharp variations in velocity and temperature close to the stenosis to ensure that the results can be repeated.

**Table 2:** Mesh characteristics summary.

Property	Value
Ratio area of element	0.05436
Mesh area	107.1 mm^2^
Minimum element quality	0.2282
Average element quality	0.7879
Quadrilateral entities	432
Mesh vertices	2281
Triangle elements	3424
Number of elements	3856
Edge elements	272
Vertex elements	14

In Table 3, the boundary has a smaller curvature factor (0.25) than the domain (0.30), which means that the walls of the curved stenosis, where flow gradients are highest, are more clearly defined. With a maximum growth rate of 1.15, the sizes of elements next to each other cannot vary by more than 15%. This makes sure that the change from the fine mesh at the stenosis to the larger elements farther away is smooth and steady. These settings, along with the fact that the independence study showed convergence at about 10,000 elements, show that the “fine” mesh of 3856 elements is sufficient, as well as that the answer is not a result of the grid resolution.

**Table 3:** Mesh sizes and settings.

Property	Value
Geometric entity level	domain and boundary
Curvature factor	0.3 (domain), 0.25 (boundary)
Minimum element size	0.016
Maximum element size	0.36
Maximum element growth rate	1.15

However, the simulations established two critical, conflicting effects driven by the nanoparticle *ϕ*. First, thermal performance is intensely enhanced at high concentrations (*ϕ* = 15*%*), leading to the thinnest thermal boundary layer and exploiting heat flux, which is valuable for microcooling applications but is rendered nearly independent of variations in Reynolds number. Second, flow stability is critically compromised by growing viscosity. Although the V_Max_ is constantly produced at the throat, the rise in *ϕ* significantly induces a transition from a parabolic to a blunt, plug‐like velocity profile. Momentously, the high viscosity intensifies flow instability, leading to extensive flow separation and the formation of large recirculation zones instantly downstream of the narrowing, particularly at high Reynolds numbers.

## 5. Biocompatibility, Toxicological Considerations, and Safe Dosing Bounds

Although the present study establishes the hemodynamic and thermal consequences of Ag–Au–Cu nanoparticle loading, the clinical translation of this trihybrid platform requires explicit consideration of the potential adverse biological effects of each nanoparticle species.

Cu nanoparticles are known to generate ROS through Fenton‐type reactions upon dissolution in biological fluids. At high local concentrations, ROS production can exceed the antioxidant capacity of endothelial cells, causing oxidative membrane damage, mitochondrial dysfunction, and apoptosis. Studies report dose‐dependent hepatotoxicity and nephrotoxicity for Cu nanoparticles at systemic concentrations exceeding 0.1 mg/kg in rodent models. The homogeneous model assumes uniform values of *ϕ*_*T*_. However, as discussed in the Section 4.4, margination and near‐wall enrichment at *ϕ*_*T*_ = 15*%* could generate local Cu concentrations at the endothelial surface substantially exceeding the bulk‐averaged value, amplifying cytotoxic risk. Ag nanoparticles release Ag^+^ ions in aqueous biological environments, which disrupt bacterial DNA replication and cell membrane integrity on the basis of their antimicrobial efficacy. However, the same ion‐release mechanism is cytotoxic to mammalian endothelial cells at elevated doses. Chronic Ag nanoparticle exposure is associated with argyria, inflammatory cytokine upregulation, and endothelial barrier disruption. Safe systemic exposure limits for Ag nanoparticles in humans are not yet formally established by regulatory agencies, but in vitro cytotoxicity thresholds for human umbilical vein endothelial cells (HUVECs) have been reported in the range of 10–50 *μ*g/mL, concentration levels that could be approached in the near‐wall margination zone at *ϕ*_*T*_ = 15*%* in the present geometry. Au nanoparticles are the most biocompatible of the three species. Their chemical inertness under physiological conditions, combined with well‐established surface functionalization chemistry, makes them the safest component of the trihybrid platform. Nevertheless, size‐dependent accumulation in the liver and spleen following intravenous administration has been documented, and long‐term clearance kinetics remain an active area of clinical investigation.

Collectively, these considerations imply that the *ϕ*_*T*_ = 15*%* case examined in this study, although computationally instructive as a boundary condition, represents a loading that requires stringent localized delivery protocols rather than systemic intravenous administration, to prevent off‐target organ exposure. The *ϕ*_*T*_ = 3*%* case most closely approximates a systemically safe loading for the metallic nanoparticle species considered, whereas *ϕ*_*T*_ = 9*%* may be appropriate for localized peristenotic delivery with appropriate surface functionalization to reduce nonspecific endothelial binding.

## 6. Novelties and Research Prospects

This study utilizes a trihybrid nanofluid composed of Ag, Au, and Cu. This nanofluid combines the antimicrobial properties of Ag (ion release that disrupts bacterial DNA/cell walls), the thermal conductivity and ROS generation of Cu (to induce oxidative stress in pathogens), and the biocompatibility, chemical inertness, antiaggregation characteristics, and SPR of Au (for targeted PTT, bioimaging, and targeted PTT, PTT, and SPR) with Cu′s advantage in generating ROS. This trimetallic platform has potential uses in medicine, such as selectively killing cancer cells with little damage to healthy tissues, as well as in improving thermal performance.

When the volume fractions of nanoparticles rise (*ϕ* = 3*%*–15*%*). This directly links nanofluid rheology, poststenotic flow instability, and cardiovascular risk. The stenosed throat has the fastest speed, but the flow separation and recirculation get stronger after the stenosis. When volume fraction (*ϕ*) increases a lot, the thermal conductivity also goes up, making heat transfer almost unaffected by flow velocity using different Reynolds numbers (600–1800). It demonstrates that thermal therapies can be applied to arteries that are too narrow. This study focuses on controlling the viscosity by using different nanoparticles in the blood flow to control poststenotic flows and lower the risk of cardiovascular problems, which could be deadly. This helps to move forward the field of precision nanomedicine.

To improve physiological realism, future work should refine viscosity and thermal conductivity formulations to incorporate shape‐dependent factors for more precise transport phenomena modeling. Transitioning to 3D simulations is essential to better characterize nonaxisymmetric flow patterns, including detailed circulation and recirculation zones in stenosed segments. Moreover, integrating MHD effects could evaluate the potential for magnetically guided delivery of trihybrid nanodrugs to stenotic regions, advancing targeted therapies in nanomedicine.

The present 2D steady‐state model establishes a computational baseline that should be extended in the following directions.

i. Non‐Newtonian rheology: The Newtonian assumption underestimates viscosity at the near‐zero core shear rates observed at high values of *ϕ*_*T*_. The Casson or Carreau–Yasuda model should be implemented to capture physiological shear‐thinning behavior accurately.
ii. Pulsatile inflow: Steady inlet conditions cannot capture transient recirculation dynamics during diastolic deceleration. A physiological Womersley velocity waveform should be imposed to evaluate how recirculation zones grow and collapse across the cardiac cycle.
iii. 3D patient‐specific geometry: The 2D domain cannot resolve azimuthal asymmetry, Dean vortices, or secondary recirculation. Patient‐specific coronary geometries reconstructed from CT imaging should be used to compute clinically relevant indices, including oscillatory shear index and fractional flow reserve.
iv. MHD extension: An external transverse magnetic field incorporated via a Lorentz body force term would enable evaluation of magnetically guided nanoparticle targeting, potentially increasing therapeutic residence time in the poststenotic zone while reducing flow instability.
v. Unequal species partitioning: The enforced equal partitioning (*ϕ*_*A* *g*_ = *ϕ*_*A* *u*_ = *ϕ*_*C* *u*_) should be relaxed. By optimizing each species fraction separately, we could find the mixtures that maximize Au‐SPR photothermal effectiveness and Ag antibiotic activity while minimizing Cu‐driven viscosity increase and circulatory instability.
vi. Experimental validation: Micro‐PIV measurements in stenosis microfluidic phantoms with fluorescently tagged nanoparticles should validate the velocity and temperature fields before clinical translation.

## 7. Conclusions

This study computationally investigated the hemodynamics and heat transfer of an Ag–Au–Cu trihybrid nanofluid in a 2D idealized stenosed artery geometry using the finite element method, systematically varying nanoparticle volume fraction (*ϕ*_*T*_ = 3*%*, 9*%*, 15*%*) and Reynolds number (Re = 600, 1200, 1800) across three time steps (*t* = 0.1, 1, 2 s). The principal conclusions, stated in physical terms, are as follows:

### 7.1. Velocity and Viscosity Trade‐Off

Increasing *ϕ*_*T*_ from 3% to 15% raises effective nanofluid viscosity (Brinkman model) and reduces peak plateau velocity in the stenosis by approximately 31% at all Reynolds numbers (e.g., from 2.46 to 1.70 m/s at Re = 1800, demonstrating that nanoparticle loading directly attenuates the inertial driving capacity of blood flow through the constriction.

### 7.2. Plug‐Like Flow Transition

Higher *ϕ*_*T*_ progressively flattens the velocity profile from parabolic (*ϕ*_*T*_ = 3*%*) to blunt, plug‐like (*ϕ*_*T*_ = 15*%*), in which velocity gradients are confined exclusively to a thin near‐wall boundary layer. This transition is mechanistically driven by accelerated cross‐sectional momentum diffusion in the more viscous nanofluid.

### 7.3. Post‐Stenotic Flow Instability

The plug‐like flow profile paradoxically intensifies poststenotic recirculation. The near‐uniform plug decelerates abruptly upon exiting the constriction, creating an adverse pressure gradient that the thin near‐wall layer cannot resist, producing the largest and most spatially persistent recirculation zones at (*ϕ*_*T*_ = 15*%*) and Re = 1800. Flow separation first appears at Re = 1200 for (*ϕ*_*T*_ = 15*%*) but is absent at the same Re for (*ϕ*_*T*_ = 3*%*), directly demonstrating the concentration‐dependent onset of hemodynamic instability.

### 7.4. Thrombogenic Shear Rate

Localized peak shear rates at the stenosis geometric transitions exceed the platelet activation threshold of 5000 s^−1^ at Re = 1800 and Re = 1200 for all three volume fractions, identifying these transition zones as primary sites of mechanically elevated thrombogenic risk. Increasing *ϕ*_*T*_ reduces peak shear rate by approximately 29*%* (from 24,000 to 17,000 s^−1^ at Re = 1800) but does not eliminate threshold exceedance at high Reynolds numbers.

### 7.5. Thermal Conductivity Enhancement and Flow‐Independent Heat Transfer

Increasing *ϕ*_*T*_ from 3% to 15% enhances the peak conductive heat flux by approximately 18% (from 7600 W/m^2^ to 9000 W/m^2^ at Re = 1800) and renders the temperature distribution effectively independent of Reynolds number at (*ϕ*_*T*_ = 15*%*), as confirmed by the near‐identical isothermal contours across Re = 600–1800. This result establishes that at sufficiently high nanoparticle loading, conductive transport dominates over advection, enabling consistent thermal dose delivery regardless of local flow velocity, a key enabler for nanofluid‐assisted PTT.

### 7.6. Critical Therapeutic Trade‐Off

Higher *ϕ*_*T*_ simultaneously improves thermal performance (beneficial for PTT) and worsens poststenotic flow instability. The present results bound but do not uniquely determine the optimal values of *ϕ*_*T*_. A value in the range (*ϕ*_*T*_ = 3*%*–9*%*) is recommended for systemic or peristenotic delivery to balance therapeutic thermal enhancement against hemodynamic safety, with (*ϕ*_*T*_ = 15*%*) reserved for localized intratumoral administration protocols.

### 7.7. Quasisteady State

All parametric cases reached quasisteady state by *t* = 1 s, confirming that the reported velocity, shear rate, and thermal fields are representative of the stable physiological flow regime and are not transient artifacts.

**Nomenclature**
**ρ**
: density, kg/m^3^
**u**
: velocity vector, m/s
**t**
: time, s
**p**
: pressure, Pa
**I**
: identity tensor
**K**
: stress tensor, Pa
**μ**
: dynamic viscosity, Pa·s
**F**
: body force vector, N/m^3^
**g**
: gravity vector, m/s^2^
**T**
: temperature, K
**C**_**p**_
: specific heat capacity, J/(kg·K)
**q**
: heat flux vector, W/m^2^
**k**
: thermal conductivity, W/(m·K)
**ϕ**
: nanoparticle volume fraction
Subscript **f**
: base fluid
Subscript **s**
: solid nanoparticle
Subscript **t** **h** **n** **f**
: Trihybrid nanofluid
Re: Reynolds number
Pr: Prandtl number
Nu: Nusselt number
Pe: Péclet number

## Author Contributions

**Muhammad Sajjad Hossain:** conceptualization, formal analysis, project administration, writing—review and editing. **Mashrur Mahfuz:** data curation, software, visualization, validation, writing—original draft. **Mahtab Uddin:** methodology, formal analysis, investigation, writing—review and editing. **Ashek Ahmed:** literature review, data curation, writing—original draft. **Kazi Ekramul Hoque:** formal analysis, writing—review and editing. **Golam Mostafa:** literature review and investigation.

## Funding

No funding was received for this manuscript.

## Disclosure

Material is reproduced from an open‐source article.

## Ethics Statement

The authors have nothing to report.

## Consent

The authors have nothing to report.

## Conflicts of Interest

The authors declare no conflicts of interest.

## Data Availability

Data sharing not applicable to this article as no datasets were generated or analysed during the current study.
